# Caregiver or Playmate? Fathers’ and mothers’ brain responses to ball-play with children

**DOI:** 10.3758/s13415-024-01237-1

**Published:** 2024-12-05

**Authors:** Dorukhan Açıl, Lara M. C. Puhlmann, Lars O. White, Pascal Vrticka

**Affiliations:** 1 Max Planck Institute for Human Cognitive and Brain Sciences, Leipzig, Germany; 2 Department of Child and Adolescent Psychiatry, Psychotherapy, and Psychosomatics, Leipzig University, Leipzig, Germany; 3 Leibniz Institute for Resilience Research, Mainz, Germany; 4 Department of Clinical Child and Adolescent Psychology and Psychotherapy, University of Bremen, Bremen, Germany; 5 Psychologische Hochschule Berlin, Berlin, Germany; 6 Department of Psychology, University of Essex, Colchester, United Kingdom

**Keywords:** Cyberball, Father-child, fMRI, Mother, Play, Social exclusion, Involvement

## Abstract

Parents and children often engage in joint play—a domain where mothers and fathers are thought to exhibit disparate behaviors and impact child development via distinct mechanisms. However, little is known about the neural substrates of mother-child and father-child play. In this fMRI study, we sampled the brain activation of parents of preschoolers (*N* = 88) during a novel event-related adaptation of the virtual ball-tossing game “Cyberball.” Mothers (*N* = 40) and fathers (*N* = 48) played “Cyberball” ostensibly with their own and an unrelated child, who consecutively included, excluded, and reincluded parents. We found that overall, exclusion yielded comparable neural activations in mothers and fathers associated with mentalizing, saliency, and emotion processing. We also observed a parent gender effect in several brain areas. While mothers exhibited increased reward- and attention-related activity during inclusion, fathers displayed increased mentalizing-related activity during exclusion. Furthermore, we tested parents’ response to reinclusion, which revealed a selective decrease in reward-related activity. Finally, exploratory analyses showed that parental involvement was positively correlated with parental brain activity within attention- and mentalizing-related areas during inclusion, as opposed to other game phases, and that an anxious parenting style was associated with increased neural sensitivity for game events involving their own child. Overall, our study elucidates the common and distinct neural networks that mothers and fathers engage during play interactions with their children, supporting theories that postulate only a partial differentiation of paternal and maternal parenting systems.

**Supplementary information:**

The online version contains supplementary material available at 10.3758/s13415-024-01237-1.

## Introduction

Parents are often their child’s first playmates. Play activity, including parental involvement therein, manifests across many species and typically figures prominently during childhood (Biben & Suomi, 1993). Play is thought to serve crucial evolutionary functions that enhance fitness of both the individual and group as a whole (Bekoff, 1984). It thus creates a safe “as-if” arena in which the child’s emotional, cognitive, language, and social skills are practiced and stimulated (Lillard, 2017; Smith, 2010). Crucially, the child may also capitalize on the parent’s involvement in their play world. The asymmetrical nature of the parent-child relationship offers the child a unique pedagogical scaffold for navigating an array of different emotions and situations (Ahnert et al., 2017; Fonagy et al., 2007; Majdandžić et al., 2018).

Interestingly, parent-child play is one of the few domains of parenting in which mothers and fathers display different behavioral tendencies (Paquette & St. George, 2023; Vallotton et al., 2020). During play interactions, mothers tend to structure, empathize, and show more attunement to the child, whereas fathers prefer more physical elements, challenge the child, and are more spontaneous, parent-centered, and intrusive (John et al., 2013; Vallotton et al., 2020). In this regard, father-child interactions are thought to contribute to social competence, emotion regulation, and resilience (Amodia-Bidakowska et al., 2020; Feldman, 2023; StGeorge & Freeman, 2017) and to protect children against anxiety (Majdandžić et al., 2018), internalizing and externalizing symptoms (Ahnert et al., 2017; Feldman & Shaw, 2021). However, to date, the parental neural mechanisms that orchestrate such parent-child play interactions remain understudied. While recent work shows how fathers’ play behavior might be associated with neural responses to their children’s faces (Mascaro et al., 2017), little or no work leverages interactive paradigms to directly examine parental neural correlates of parent-child play as it unfolds in real time.

To this end, we adapted a virtual ball-tossing game (“Cyberball”; Williams et al., 2000) to create an event-related parent-child fMRI version that simulates a parent-child play episode. Traditionally, Cyberball ostensibly connects participants to two unfamiliar online game partners who are actually computer-generated. They initially include, before excluding**,** participants in the course of the game. Copious work using Cyberball across the past two decades has shown exclusion-related activation of neural regions as part of the default-mode network (Mwilambwe-Tshilobo & Spreng, 2021) as well as areas involved in experiencing and regulation of aversive emotions (Seeley, 2019), self-evaluative processing (Vijayakumar et al., 2017), and “social pain” (Eisenberger, 2015), among others. Thus, the Cyberball task elicits key neurocognitive-affective processes that may prove highly relevant for typical parent-child play interactions. Besides, as a triadic ball-play, Cyberball has further potential to yield increased activation in the following brain networks associated with parenting: (1) the insular-cingulate “saliency or empathy network” (including the anterior insula and dorsal anterior cingulate cortex, amongst others), (2) the temporoparietal “mentalizing network” (including the posterior cingulate cortex and temporoparietal junction, amongst others), (3) the “emotion-regulation network” (including the dorsolateral and orbitofrontal prefrontal cortices, amongst others), (4) the “embodied-simulation network” (including the intraparietal lobule and supplementary motor area, amongst others), (5) the “executive network” (including the prefrontal cortices, amongst others), and finally (6) “arousal-, attention-, and motivation-related circuits” (for more details, see Feldman et al., 2019; Swain et al., 2014b). 

Cyberball is also a suitable paradigm to compare mothers’ and fathers’ brain activations in the play context. The available research on play situations suggests that mothers’ and fathers’ parenting neurobiology show substantial similarities, with some consistent differences (Feldman et al., 2019). In general, fathers tend to show stronger activations in cortical areas involving social cognition and mentalizing as a response to child stimuli, whereas mothers show stronger activations in arousal- and motivation-related circuits (Abraham & Feldman, 2022). Furthermore, fathers’ parenting neurocircuitry seems to exert greater plasticity as a response to their caregiving experiences, induced by the exposure to infant or pregnancy cues (Abraham et al., 2014; Atzil et al., 2012). Thus, Cyberball is nicely suited to study whether such differences extend to the play domain with its exclusion and inclusion dynamics that reenact approach, separation, and reunion behaviors inherent to every parenting interaction.

Thus far, only two neuroimaging studies have examined parent-child dyads in Cyberball. A previous ERP study (Sreekrishnan et al., 2014) reported that both mothers and children exhibited more pronounced P2 and left-frontal positive slow-waves when rejected by one another (compared with rejection by strangers). Furthermore, in an fMRI study (van den Berg et al., 2018), parents’ and their (mostly adult) offspring’s neural responses to exclusion by one another elicited significantly greater activity in the ACC (relative to exclusion by strangers). Taken together, evidence suggests that parent-child dyads could be particularly sensitive to rejection by each other as opposed to strangers. Crucially, however, no work to date compares mothers’ and fathers’ neural responses to inclusion and exclusion from play interactions with their own children at a younger age when play figures particularly prominently in development.

The present study was designed to investigate both mothers’ and fathers’ neural activity during real-time parent-child play by using Cyberball. Parents engaged in the task ostensibly with their own preschool-aged child and another unrelated child. To our knowledge, ours is the first study to directly compare mothers’ and fathers’ neural responses in an fMRI interaction paradigm, particularly in a play context. We focused specifically on parents of preschool-age children, because many forms of play as well as parental involvement in play activity peak in the preschool years before declining thereafter (Pellegrini et al., 2007; Amodia-Bidakowska et al., 2020).

Most studies using Cyberball to date used block designs by averaging neural activity across phases, usually involving inclusion and exclusion. However, tapping into different dynamics of a play episode between parents, their child, and an unrelated child necessitated an event-related Cyberball design (Gunther Moor et al., 2012; Preller et al., 2016; Schulz et al., 2022). Our event-related paradigm allowed us to distinguish the events involving one coplayer from those involving the other coplayer and therefore was tailored to dissect the neural activity related to several play episodes between different combinations of agents. Thereby, our task also made it possible to compare directly the parental brain activity related to the own versus an unrelated child. Finally, we added a reinclusion condition for the first time in an event-related fMRI design. In sum, this procedure allowed us to distinguish mothers’ and fathers’ neural responses one by one to (1) playing with, (2) being excluded by, and (3) being reincluded by (a) their own child and (b) an unrelated child.

## Methods

### Sample

A total of 91 parents participated in this study. One participant had to be excluded because of excessive movement during scanning (i.e., continuous motion exceeding 2.5 mm in x, y, and/or z translation) and two because of too many behavioral errors during Cyberball. The final sample, therefore, included 88 participants (*N* = 48 fathers; see Table 1 for demographics & Supplementary Materials for information on recruitment). Ethical approval for the study was obtained from the local ethics committee. Participants declared their written informed consent for themselves and their children prior to the study and received monetary compensation (parents) and a small gift (children) for their participation.

**Table 1:** Demographic information

Sample characteristics	Overall	Mothers	Fathers	Between-group comparisons	
				Test statistic	*p*
Mean participant age in years (*SD*)	38,35	36,81	39,94	*t*(86) = 2.66	.009*
% left handedness	0	0	0		
Median school education	Bachelor degree	Bachelor degree	Bachelor degree	*U* = 2073	.55
% of daughters	49%	55%	44%	χ^2^(1) = 1.11	.29
Child age	5,57	5,37	5,73	*t*(86) = 4.51	<.001***

### Procedure

The study was conducted at the Max Planck Institute for Human Cognitive and Brain Sciences, Leipzig, Germany in a testing session in which parents and their children participated together. Upon arrival, parents and their children were led to a room where the child would be tested. Parents were led to believe that their children would participate in the computerized ball-tossing game, Cyberball, utilizing a computer present in this room. To reinforce this perception, we placed a computer screen showing the Cyberball task, a real baseball glove, and a ball on the table. Parents were then taken to the MRI facilities, where they underwent MRI scanning, comprising a structural sequence and two functional scans, one of which was the Cyberball task. Before commencing with Cyberball in the scanner with parents, we had them play a practice session “on their own” without the children ostensibly connected, followed by a fake connection sequence linking up all three computers to start the game. After scanning, parents were informed about the experimental deception, and they signed a document that their data could be analyzed nonetheless. While parents were undergoing MRI scanning, children participated in another assessment relevant to the project not considered here. Pictures of children that were shown to parents during the Cyberball task were taken previously at another timepoint.

### Cyberball

During the collection of functional brain images, parents performed an event-related version of the Cyberball paradigm (Williams et al., 2000), programmed in Presentation^®^ (Version 18.0, Neurobehavioral Systems, Inc., Berkeley, CA). Parents were led to believe that they were connected with their own child playing in the other room, as well as another unrelated child also present in the building at the time.

**Fig. 1:**
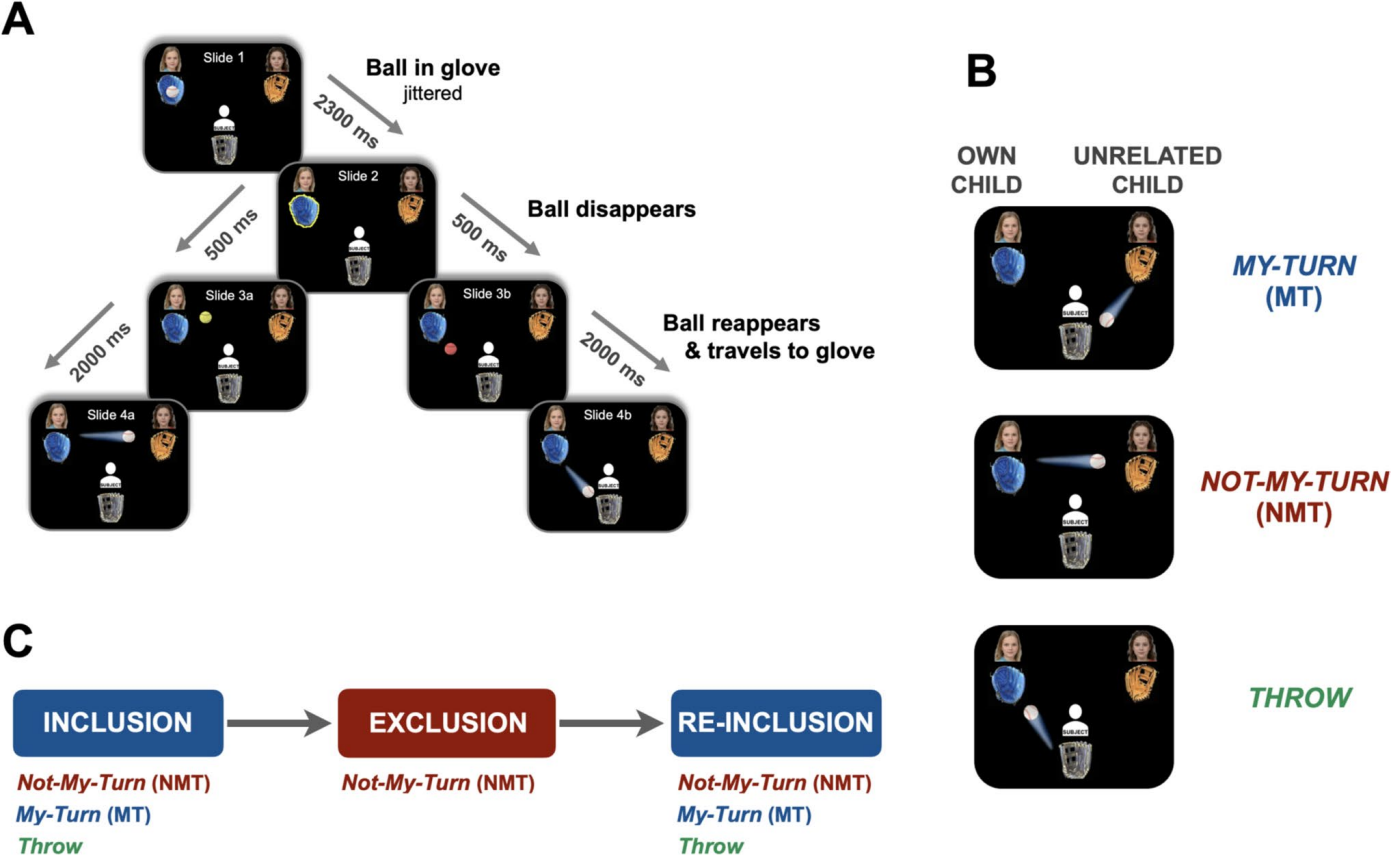
Cyberball task. **A.** How static events were introduced in a dynamic paradigm. **B.** Each task event with two familiarity conditions (as coplayers): own and unrelated child. **C.** Task flow with each task event that is embedded in each main task conditions, aka game phases

This Cyberball design (Fig. 1) was tailored to an event-related analysis by imposing static events on a dynamic paradigm via transient disappearance and reappearance of the ball (Fig. 1A). Hence, it allowed us to compare neural responses during the three main conditions representing the principal game phases—i.e., Inclusion, Exclusion, Reinclusion—as well as three within-condition events : i.e., *my-turn* (MT) when parents received the ball; *not-my-turn* (NMT) when parents observed the ball to be thrown between the two children; and *throw* when parents clicked a button to throw the ball either to their own or the unrelated child (Fig. 1B). After a short practice session without connection to other coplayers, the game started with the inclusion phase within which all players received the ball at equal rates (~1/3 of all tosses and ~15 times each). The inclusion phase was followed by an exclusion phase during which the two children tossed the ball almost exclusively among each other, thereby excluding the parent (who still very sporadically received the ball to draw attention to the game). The game was programmed to proceed into a reinclusion phase, during which children began tossing the ball to the parent again at equal rates (Fig. 1C). After scanning, parents were asked whether they had any doubts about the genuineness of the paradigm by using a single question with Likert scale answer options ranging from 1 = “completely disagree” to 5 = “completely agree.” Their mean response was 2.96.

### Exploratory self-report measures

#### Parental responsibility scale

An adapted version of the Parental Responsibility Scale (PRS; McBride and Mills, 1993) was used to measure the degree to which mothers and fathers took responsibilities in parenting tasks. The scale lists 14 common childcare tasks that parents are expected to report their involvement in (e.g., “spend special time at bedtime”; “buy child’s toys”). Crucially, the PRS encourages parents to evaluate who remembers, plans, and schedules the tasks, rather than only who ends up performing them. Parents in our study filled the task on their own. The Likert scale for the father version ranged from 1 = “Mother always responsible” to 5 = “Father always responsible.” The mother version was formulated in the opposite direction. Therefore, higher scores always indicated stronger involvement of the parent who filled out the questionnaire. We averaged the scores of the 14 items, calling it “Parental Involvement” hereafter. Internal consistency was excellent with Cronbach’s alpha at .91. Four participants (3 fathers and 1 mother) did not provide any answers to this questionnaire; therefore, we used a sample of 84 in any analyses that included Parental Involvement.

#### Caregiver experiences questionnaire

A validated four-factor version of the Caregiver Experiences Questionnaire (CEQ; Røhder et al., 2019) was used to measure parents’ attitudes and feelings about caregiving experiences. Items were translated to German for this study. The questionnaire has 40 items, which constitute the following four factors: Delight/Enjoyment (e.g., “I enjoy being with my child when s/he is learning”); Heightened (e.g., “I am lonely when my child and I are separated”); Helplessness (e.g., “There are a lot of times when I cannot control or restrain my child”); and Role reversal (e.g., “My child goes out of his way to be sensitive and tuned in to me and others”). All responses are given on a Likert scale ranging from 1 = “not at all characteristic” to 5 = “very characteristic”). We only included the Delight and Heightened subscales in this study, because they are the most relevant constructs for the inclusion and exclusion related play interactions. Delight is composed of 17 items that had good internal consistency, Cronbach’s alpha = .76. Heightened included 5 items also with a good internal consistency, Cronbach’s alpha = .75. Two participants (2 fathers) did not provide any responses for this questionnaire; therefore, we used a sample of *N* = 86 in all analyses that included the CEQ subscales. We refer to these subscales as “Delightful Parenting” and “Heightened Parenting” throughout the remainder of the paper. Delightful Parenting refers to parenting experiences involving enjoyment/pleasure and to a more positive parent-child representation as carried by the parent. Heightened Parenting refers to a more anxious parent-child representation of the parent accompanied by desire for excessive proximity with the child.

### fMRI data acquisition

MRI data were collected on a 3T Siemens Skyra scanner with a 32-channel head coil. Structural scans were acquired using a T1-weighted Magnetization Prepared Rapid Gradient Echo (MPRAGE) sequence (TR = 2300 ms, TE = 2.98 ms, flip angle = 9°, FoV = 256 mm, voxel size: 1 × 1 × 1 mm, 176 slices). Functional scans were obtained using T2*-weighted gradient-echo planar imaging (GE-EPI) with multi-band acceleration (acceleration factor 3; TR = 2000 ms, TE = 22 ms, flip angle = 80°, FoV = 204 mm, voxel size: 2.5 × 2.5 × 2.5 mm, interslice gap: 0.25 mm, 60 slices (interleaved; Feinberg et al., 2010; Moeller et al., 2010).

### Data analysis

#### Behavioral analyses

While the game behavior of the two child coplayers was scripted, parents were free in their choice of either passing the ball to their own or the unrelated child. To test whether there were any differences in the number of ball throws to each child, we conducted a repeated-measures ANOVA with the number of ball throws as the dependent variable, and child familiarity (own vs. unrelated), game phase (inclusion vs. reinclusion), and parent gender as independent variables. We also included genuineness ratings in the model to control for any possible effects.

#### fMRI data analyses

fMRI data were preprocessed and analyzed by using Statistical Parametric Mapping (SPM12; version 7771; Wellcome Department of Imaging Neuroscience Group, London, UK). The first five volumes of the functional time series were discarded to allow for T1 equilibration, and all other images were visually inspected for potential signal loss. The pre-processing was completed by using standard procedures of slice time correction, realignment with a rigid body transformation, and co-registration. The pre-processed volumes were normalized to the Montreal Neurological Institute (MNI) template with a 12-parameter affine and nonlinear transformation (ICBM152; dimensions: 91 × 109 × 91; voxel size: 2 mm^3^). At last, spatial smoothing was applied using an 8-mm Full Width at Half Maximum (FWHM) Gaussian kernel.

We included 15 event types as regressors in the statistical model. These included six regressors for inclusion (3 events x 2 familiarity conditions), the same six for reinclusion, and two regressors (1 event [NMT] x 2 familiarity conditions) for the exclusion phase. One additional regressor was included for erroneous trials (i.e., parents not responding during throw trials or responding during NMT trials). Single-subject design matrices (first-level analysis) were created with the onsets of these fifteen event regressors with zero durations that were convolved with the canonical hemodynamic response function, additionally including the six motion parameters obtained during realignment. Model estimation also included a high-pass frequency filter (128 s), and corrections for autocorrelation between scans were applied to the time-series data.

Using these event regressors, we defined the following four contrasts to test the main task effects. As preregistered, (1) “NMT > MT during Inclusion” and (2) “NMT during Exclusion > NMT during Inclusion” were used to test exclusion effects. In addition, an important exclusion contrast that has been used in previous event-related Cyberball studies (Gunther Moor et al., 2012; Preller et al., 2016; Schulz et al., 2022), but that was not included in our preregistration, was also investigated exploratively: (3) NMT during Exclusion > MT during Inclusion. To test reinclusion effects, we used the interaction contrast (4) NMT vs. MT for Inclusion vs. Reinclusion. These four contrasts were computed first by combining the event regressors for own and unrelated children to test effects without considering familiarity. Followingly, additional contrasts were computed to test for familiarity effects by contrasting the event regressors for own versus unrelated children for each task contrast separately (except for the Reinclusion contrast #4, because it was not possible to define a three-way interaction in SPM without changing the first-level task design). Due to the novelty of our design, we preregistered several additional task contrasts, the results of which we report in the Supplementary Materials for the sake of readability and conciseness.

The above single-subject contrasts were subsequently used to generate a random-effects group-level whole-brain analysis (based on one-sample *t*-tests) across all participants to examine task-induced brain activation. All group-level analyses were conducted with *p* < .001 uncorrected on the voxel level and a subsequent FWE-correction of *p* < .05 at the cluster level. Finally, to test the effects of parents’ and children’s gender separately, we conducted two-sample *t*-tests with the same analytic approach. In the case of significant effects in a two-sample *t*-test on the whole-brain level, we extracted raw activation (beta) values to subject them to a post-hoc repeated measures analysis of variance (ANOVA) for further decomposition.

Furthermore, we tested associations between the three self-report parenting measures (Parental Involvement, Delightful Parenting, and Heightened Parenting) and the preregistered contrasts along with the additionally included exclusion contrast (#3) mentioned above as post-hoc exploratory analyses. To this end, we included the three self-report measures separately as regressors in whole-brain SPM models. In the case of significant activity, we extracted beta values from selected regions of interest (ROIs) to test for parent gender effects with a post-hoc ANOVA by correcting for the number of comparisons performed using Bonferroni corrections.

 Matlab® 2022b and the Canlab toolbox (https://github.com/canlab) were used to visualize the brain images. R (version 3.6.3.; R core Team, 2020) was used for further image production.

## Results

### Behavioral findings

There was a significant main effect of child familiarity, *F*(1,85) = 5.11, *p* = .026 such that parents threw the ball more frequently to their own child (*M* = 15.78) than to the unrelated child (*M* = 13.76). Please refer to the *Supplementary* Figure S9 for an illustration. There were no significant main or interaction effects pertaining to the game phase or parent gender.

### Whole-brain effects of task conditions

In the first step, we tested the task effects using eight event-related contrasts across all participants (i.e., mothers and fathers) by combining the effects of the two familiarity conditions (i.e., own and unrelated child). We then followed up on each contrast by testing for a possible effect of familiarity by contrasting brain activity relating to own versus unrelated child. For the sake of brevity, we only report the results from four of these eight contrasts here. Please refer to the Supplementary Materials for all remaining results. Below, we describe the significantly active clusters from a global networks perspective (Uddin et al., 2019)

#### Exclusion

##### Not-my-turn (NMT) > My-turn (MT) within Inclusion

**Fig. 2:**
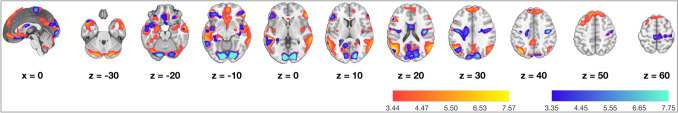
Whole brain activation differences (*N* = 88) for Exclusion > Inclusion across two contrasts: *NMT* > *MT* during Inclusion (in red/yellow), and *NMT* Exclusion > *MT* Inclusion (in blue). Random-effects (one-sample *t*-test), *p* < .001 uncorrected at the voxel level and *p* < .05 FWE-corrected at the cluster level

NMT compared with MT events during the Inclusion phase activated three main networks: the default mode network (e.g., bilateral dorsal and medial prefrontal cortices [dmPFC; vmPFC], temporoparietal cortex [TPJ], precuneus, posterior cingulate cortex [PCC]; middle temporal gyrus [MTG]), saliency network (e.g., bilateral anterior insula [aINS], posterior insula [pINS], dorsal anterior cingulate cortex [dACC]), and areas implicated in emotion experience and regulation (Etkin et al., 2006), such as bilateral lateral prefrontal cortex (PFC), subgenual anterior cingulate cortex (sgACC), and amygdala. Additional significant clusters were observed in visual processing areas, including bilateral striate cortex, cuneus, and left fusiform gyrus; somatomotor cortices, covering bilateral precentral (preCG) and postcentral (postCG) gyri, premotor cortex, and supplementary motor area (SMA); as well as in areas such as the bilateral cerebellum, left hippocampus and left parahippocampal gyrus (Table 2; Fig. 2). We did not find any significant activation differences pertaining to familiarity in this contrast.

##### Not-my-turn (NMT) during Exclusion > My-turn (MT) during Inclusion

NMT during Exclusion compared with MT during Inclusion activated the saliency network (e.g., bilateral aINS, dACC, and pINS); the central-executive network (e.g., bilateral ventrolateral prefrontal cortex [vlPFC] and caudate nucleus); and the default mode network nodes, such as left MTG/superior temporal sulcus, left PCC (ventral), left angular gyrus (AG), and right hippocampus. Additional regions included the bilateral extrastriate cortex, cuneus, preCG (Table 3; Fig. 2). We did not find any significant activation differences pertaining to familiarity in this contrast.

##### Not-my-turn (NMT) during Exclusion > Inclusion

Whole-brain analyses for this contrast did not yield any significant clusters. We also did not find any significant activation differences pertaining to familiarity.

#### Reinclusion

##### My-turn (MT) versus Not-my-turn (NMT) events for Inclusion versus Reinclusion

**Fig. 3:**
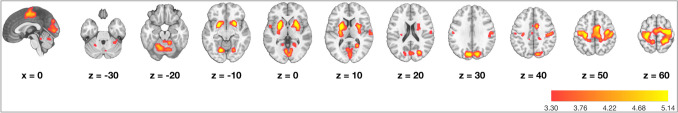
Whole brain activation differences (*N* = 88) for the interaction contrast, Reinclusion vs. Inclusion for *NMT* vs. *MT* events. It illustrates the areas that showed a significant interaction between two task conditions (Reinclusion vs. Inclusion) and two event conditions (*NMT* vs. *MT*). Random-effects (one-sample *t*-test), *p* < .001 uncorrected at the voxel level and *p* < .05 FWE-corrected at the cluster level

This contrast revealed significant effects in four clusters: A left putamen cluster that included the claustrum; a right putamen cluster along with aINS; a motor cluster containing bilateral SMA, preCG, and postCG; and an occipital cluster including bilateral cuneus, lingual gyrus that extends to bilateral cerebellum and right PCC (Table 4; Fig. 3).

**Table 2:** Whole brain activations in NMT > MT within Inclusion (*N* = 88)

Anatomical region	Hemisphere	x,y,z	*t*	*z*	*k*	*p*
Extrastriate cortex/ Lingual gyrus	R	14, -90, -6	19,01	Inf	21648	<.001
Extrastriate cortex/ Lingual gyrus	L	-12, -90, -10	17,12	Inf		
Cuneus	R	14, -86, 30	10,09	Inf		
vlPFC	L	-50, 38, -4	9,74	Inf		
Premotor cortex	L	-46, -10, 32	9,37	7,76		
Angular gyrus	L	-52, -70, 22	9,32	7,73		
Cuneus	L	-8, -88, 26	8,67	7,34		
MTG	L	-52, -38, 2	8,64	7,32		
Dorsolateral prefrontal cortex (dlPFC)	L	-54, 28, 12	8,49	7,23		
Posterior insula	R	38, -14, 20	9,64	Inf	7896	<.001
Precentral gyrus/premotor cortex	R	40, -14, 40	8,22	7,05		
SMA	R	6, -22, 64	8,18	7,03		
SMA	L	-6, -28, 74	7,87	6,82		
TPJ	R	58, -60, 22	7,61	6,64		
Angular gyrus	R	56, -64, 20	7,57	6,62		
Anterior insula	R	42, 0, -8	7,29	6,43		
MTG	R	64, -44, -6	7,02	6,23		
vlPFC	R	50, 44, -8	8,74	7,38	997	<.001
dmPFC	L	-14, 52, 38	6,66	5,97	2535	<.001
dmPFC	R	4, 44, 50	6,63	5,95		
SMA	L	-6, 24, 66	6,1	5,55		
Cerebellum	L	-6, -54, -42	5,07	4,73	301	<.001
Parahippocampal gyrus	L	-32, -38, -12	4,79	4,5	349	<.001
Fusiform gyrus	L	-40, -40, -16	4,39	4,16		
Hippocampus	L	-24, -26, -6	4,15	3,95		

*Note*. Clusters listed are significant in a whole-brain analysis (*p* < .001 uncorrected at voxel level and *p* < 0.05 FWE-corrected at cluster level, k > 20 voxels). x, y, z refer to MNI coordinates. T refers to the t-score and z the z-score at those coordinates (local maxima). K refers to the number of voxels in each significant cluster

**Table 3:** Whole brain activations in NMT during Exclusion > MT during Inclusion (*N* = 88)

Anatomical region	Hemisphere	x, y, z	*t*	*z*	*k*	*p*
Extrastriate cortex	R	14, -92, -2	15,56	Inf	5341	<.001
Extrastriate cortex	L	-12, -90, -2	14,97	Inf		
Cuneus	R	14, -84, 26	8,1	6,98		
Cuneus	L	-16, -92, 26	5,8	5,32		
PCC (ventral)	L	-14, -52, 12	5,45	5,04		
Posterior insula	R	38, -14, 20	8,28	7,09	897	<.001
Precentral gyrus	R	40, -12, 40	7,77	6,75		
Precentral gyrus	L	-6, -26, 64	7,23	6,38	1190	<.001
Precentral gyrus	R	16, -30, 76	5,99	5,46		
Precentral gyrus	L	-42, -12, 34	7,09	6,28	1598	<.001
dACC	L	-6, 2, 22	6,34	5,73		
dACC	R	6, 0, 22	5,45	5,04		
Posterior insula	L	-38, -14, 24	5,12	4,77		
Caudate nucleus	L	-22, 2, 26	4,1	3,91		
vlPFC	L	-54, 30, 8	6,45	5,81	780	<.001
Orbitofrontal cortex (OFC)	L	-24 30 -12	5,36	4,97		
MTG	L	-58, -50, -8	5,69	5,23	464	0.004
Superior temporal sulcus	L	-66, -30, 4	3,85	3,69		
Anterior insula	L	-40, 2, -12	5,67	5,22	537	0.002
STG	L	-54, -8, -14	5	4,68		
Anterior insula	R	44, 2, -12	5,62	5,18	347	0.014
STG	R	48, -6, -14	4,88	4,57		
Hippocampus	R	38, -18, -10	4,44	4,2		
Caudate nucleus	R	36, -26, -6	3,3	3,19		
OFC	R	36, 40, -10	5,57	5,14	258	0.042
Angular gyrus	L	-40, -66, 28	5,27	4,89	338	0.016

*Note*. Clusters listed are significant in a whole-brain analysis (*p* < .001 uncorrected at voxel level and *p* < 0.05 FWE-corrected at cluster level, k > 20 voxels). x, y, z refer to MNI coordinates. T refers to the t-score and z the z-score at those coordinates (local maxima). K refers to the number of voxels in each significant cluster

**Table 4:** Whole brain activations in the interaction of Inclusion vs. Reinclusion for my-turn (MT) vs. not-my-turn (NMT) (*N* = 88)

Anatomical region	Hemisphere	x, y, z	*t*	*z*	*k*	*p*
Precentral gyrus	R	28, -16, 68	7,81	6,78	7303	<.001
Precentral gyrus	L	-12, -14, 68	6,57	5,9		
Postcentral gyrus	R	28, -42, 64	6,41	5,78		
SMA	R	8, -2, 52	5,56	5,13		
Postcentral gyrus	L	-16, -46, 68	4,96	4,64		
Putamen	L	-24, 8, -4	6,96	6,19	1494	<.001
Claustrum	L	-26, -24, 14	5,28	4,9		
Putamen	R	24, 10, -6	6,31	5,71	1162	<.001
Anterior insula	R	38, 2, 14	3,67	3,53		
Lingual gyrus	L	-16, -68, -10	6,07	5,53	3656	<.001
Cuneus	L	-16, -80, 28	5,94	5,43		
Cuneus	R	12, -80, 34	5,5	5,08		
Cerebellum	L	-24, -54, -22	4,9	4,59		
PCC	R	22, -54, 8	4,76	4,47		
Lingual gyrus	R	24, -66, -2	4,63	4,37		
Cerebellum	R	14, -66, -22	4,53	4,28		

*Note*. Clusters listed are significant in a whole-brain analysis (*p* < .001 uncorrected at voxel level and *p* < 0.05 FWE-corrected at cluster level, k > 20 voxels). x, y, z refer to MNI coordinates. T refers to the t-score and z the z-score at those coordinates (local maxima). K refers to the number of voxels in each significant cluster

**Fig. 4:**
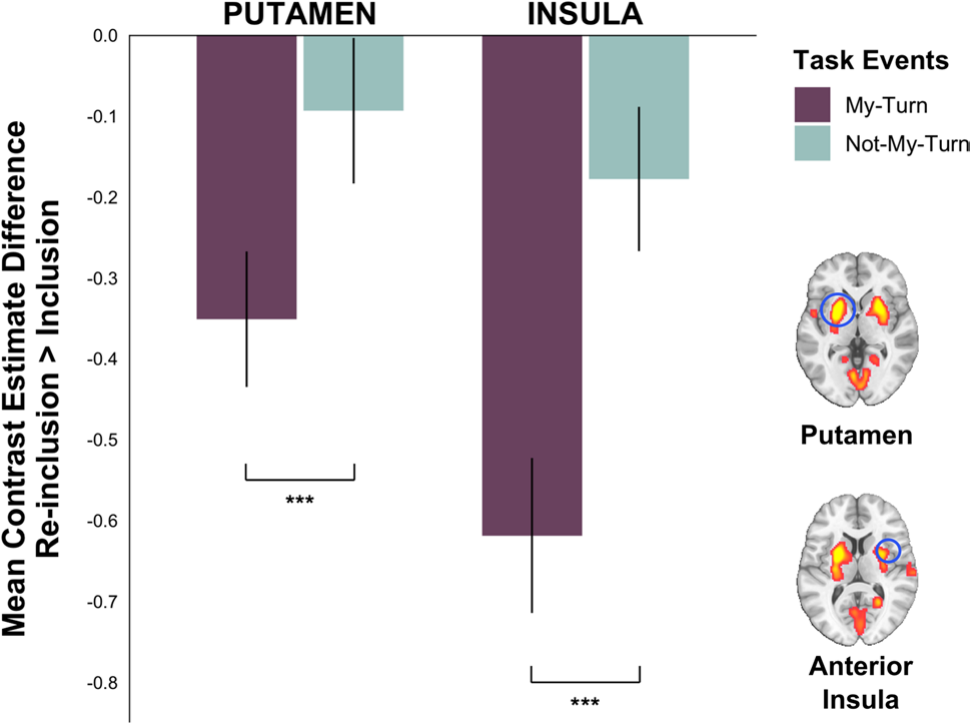
The bar plot displays the mean activation (beta) values and their standard errors (y-axis) for the contrast, Reinclusion > Inclusion across two events, *MT* (purple) and *NMT* (turquoise) in two regions (x-axis): left putamen cluster (peak voxel at [−24 8 −4], k =1494), and a 4-mm–wide sphere around the right anterior insula (peak voxel at [38 2 14]). n.s. = nonsignificant; *p < .05; ***p < .001

We extracted raw activation (beta) values from two of the above regions to better understand and illustrate this interaction pattern. The left putamen cluster (peak voxel at [−24 8 −4], k = 1494) revealed a specific decrease in MT activity during Reinclusion versus Inclusion, *F*(1, 87) = 39.215, *p* < .001, η_p_^2^ = .311. Similarly, activity from a 4-mm-wide sphere around the right aINS (peak voxel at [38 2 14]) showed reduced activity for MT events during Reinclusion versus Inclusion, *F*(1, 87) = 11.393, *p* < .001, η_p_^2^ = .116 (Fig. 4).

### Parent gender

In a second step, as it was preregistered, we explored whether mothers and fathers neurally responded to the Cyberball task differentially. To do so, we conducted additional two-sample *t*-tests on the 15 preregistered contrasts: 8 contrasts that combined familiarity conditions, and another 7 that tested for the effects of familiarity. The comparisons that revealed significant activation differences between mothers and fathers were as follows.

#### Inclusion

##### My-turn (MT) versus Not-my-turn (NMT) within Inclusion

**Fig. 5:**
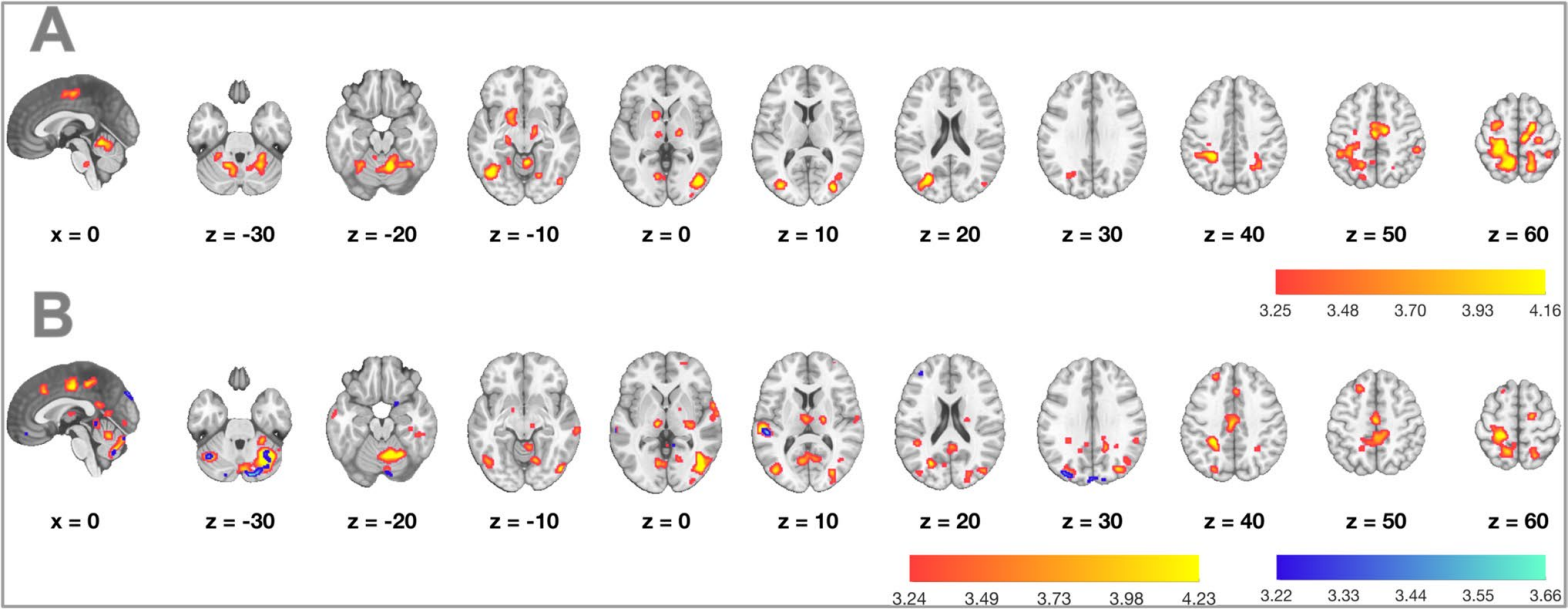
Whole brain activation differences between mothers (N = 40) and fathers (*N* = 48). Panel **A** illustrates the contrast, *NMT* > *MT* during Inclusion. Panel **B** illustrates two contrasts: *NMT* during exclusion > *MT* during Inclusion (in red/yellow) and *NMT* during Exclusion > *NMT* during Inclusion (in blue). Two-sample *t*-tests, *p* < .001 uncorrected at the voxel level and *p* < .05 FWE-corrected at the cluster level

This comparison revealed an interaction between parent gender and brain activity in a left postCG cluster covering preCG, SMA, superior parietal lobule, and intraparietal sulcus (IPS); a right SMA/middle cingulate cortex (MCC) cluster extending to preCG; a cluster covering bilateral cerebellum, fusiform gyrus and right lingual gyrus; as well as in additional areas including bilateral middle occipital gyrus (extending toward right MTG), left precuneus, and left putamen (Table 5; Fig. 5A).

**Table 5:** Whole brain activation differences for mothers (*N* = 40) vs. fathers (*N* = 48)

***not-my-turn (NMT) > my-turn (MT) during Inclusion***						
Anatomical region	Hemisphere	x, y, z	t	z	k	p
Postcentral gyrus	L	-26, -34, 58	5,1	4,76	3074	<.001
Precentral gyrus	L	-16, -12, 68	4,84	4,54		
Superior parietal lobule	L	-18, -54, 58	4,82	4,52		
Intraparietal sulcus	L	-28, -44, 36	4,62	4,35		
SMA	L	-32, -8, 64	3,96	3,78		
SMA	R	8, -20, 62	5,01	4,68	1069	<.001
SMA	L	-4, -14, 52	4,41	4,18		
Middle cingulate gyrus	R	10, -12, 50	4,34	4,11		
Precentral gyrus	R	22, -22, 66	4,02	3,84		
Cerebellum	R	24, -54, -24	4,95	4,63	1147	<.001
Fusiform gyrus	R	36, -52, -14	4,67	4,39		
Cerebellum	L	-6, -60, -14	4,02	3,84		
Lingual gyrus	R	18, -68, -8	3,97	3,79		
Middle occipital gyrus	R	42, -74, 0	4,87	4,57	549	<.001
MTG	R	42, -78, 20	3,46	3,34		
Fusiform gyrus	L	-44 -66 -12	4,82	4,52	695	<.001
Cerebellum	L	-16, -54, -28	4,16	3,96		
Middle occipital gyrus	L	-30, -72, 20	4,63	4,36	482	0.003
Putamen	L	-14, 14, -4	4,22	4,01	304	0.022
Precuneus	R	14, -58, 62	4,16	3,96	491	0.003
Superior parietal lobule	R	16, -48, 64	4,04	3,85		
***not-my-turn (NMT) during Exclusion > my-turn (MT) during Inclusion***						
Anatomical region	Hemisphere	x, y, z	t	z	k	p
Intraparietal sulcus	L	-30, -40, 36	5,55	5,11	1822	<.001
SMA	L	-18, -16, 76	4,75	4,46		
Postcentral gyrus	L	-24, -34, 58	4,74	4,45		
Precuneus	L	-6, -44, 54	4,24	4,03		
Precentral gyrus	L	-26, -20, 70	4,22	4,01		
PCC (dorsal)	Bilateral	0, -34, 48	4,18	3,97		
PCC (ventral)	L	-10, -46, 28	3,35	3,24		
Cerebellum	R	32, -56, -30	5,53	5,1	3261	<.001
Cerebellum	L	-2, -70, -38	4,82	4,52		
Cerebellum	L	-44, -60, -36	5,28	4,9	522	0,002
Extrastriate cortex	R	40, -62, 2	5,12	4,77	1046	<.001
Fusiform face area	R	46, -76, -8	4,54	4,29		
Intraparietal sulcus	R	32, -78, 34	4,2	4		
Middle occipital gyrus	R	28, -80, 10	4,05	3,86		
Cuneus	R	42, -78, 24	3,97	3,79		
Fusiform gyrus	R	52, -66, 0	3,75	3,6		
Superior occipital gyrus	L	-26, -68, 22	4,81	4,51	555	0,001
Middle occipital gyrus	L	-44, -80, 14	4,66	4,39		
STG	L	-60, -26, 10	4,58	4,32	278	0,003
Middle cingulate gyrus	Bilateral	0, -14, 44	4,44	4,2	362	0,010
***not-my-turn (NMT) during Exclusion > not-my-turn (NMT) during Inclusion***						
Anatomical region	Hemisphere	x,y,z	t	z	k	p
Cerebellum	R	6, -82, -24	3,99	3,81	267	<.001

*Note*. Clusters listed are significant in a whole-brain analysis (*p* < .001 uncorrected at voxel level and *p* < 0.05 FWE-corrected at cluster level, k > 20 voxels). x, y, z refer to MNI coordinates. T refers to the t-score and z the z-score at those coordinates (local maxima). K refers to the number of voxels in each significant cluster

**Fig. 6:**
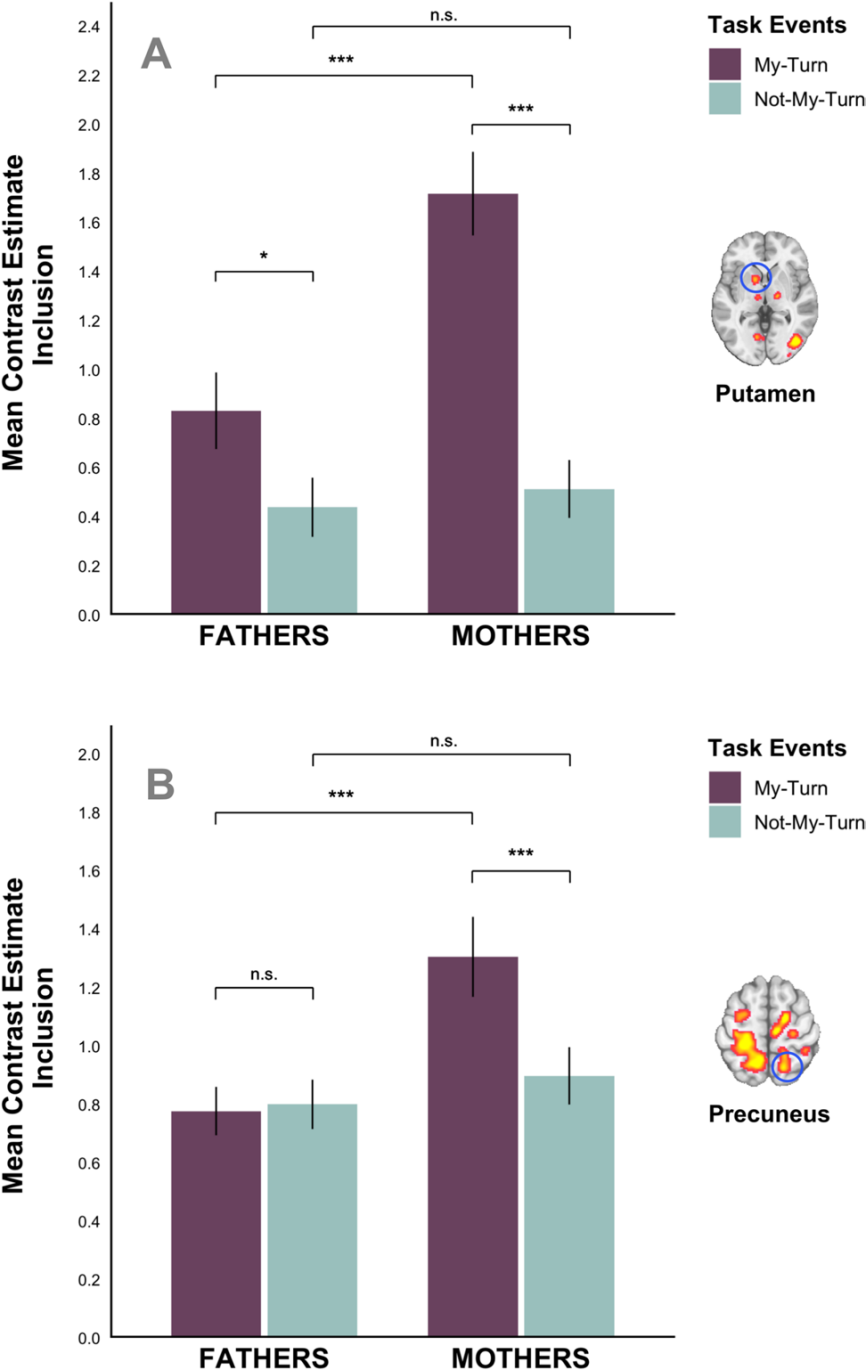
The bar plots display the mean activation (beta) values and their standard errors (y-axis) for the contrast, *MT* (purple) > *NMT* (turquoise) during Inclusion for mothers (*N* = 40) and fathers (*N* = 48; x-axis). Panel **A** illustrates the activity in a left putamen cluster (peak voxel at [−14 14 −4], k = 304). Panel **B** illustrates the activity in a right precuneus cluster (peak voxel at [14 −58 62], k = 491). n.s. = nonsignificant; *p < .05; ***p < .001

We extracted beta values from several of the above regions (i.e., left putamen, right precuneus, right dACC, right middle occipital gyrus/MTG) to better understand and illustrate this interaction pattern. Bonferroni corrected post-hoc comparisons within ROIs revealed three consistent patterns (Fig. 6; see Supplementary Table S13 for detailed descriptive statistics). i) Most consistently, mothers’ brain activity was significantly higher for MT > NMT during Inclusion in all ROIs, namely the putamen (*F*(1,86) = 70.90, *p* < .001), precuneus (*F*(1,86) = 39.35, *p* < .001), dACC (*F*(1,86) = 33.38, *p* < .001) and middle occipital gyrus/MTG (*F*(1,86) = 43.80, *p* < .001). ii) The second emerging pattern was that mothers’ brain activity was significantly higher than fathers’ brain activity for MT during Inclusion in three ROIs, namely the putamen (*F*(1,86) = 14.67, *p* < .001), precuneus (*F*(1,86) = 11.71, *p* < .001), and middle occipital gyrus/MTG (*F*(1,86) = 24.42, *p* < .001). iii) Finally, only in the putamen, fathers’ brain activity was significantly higher for MT > NMT during Inclusion, *F*(1,86) = 9.08, *p* < .001. We did not find any significant activation differences pertaining to familiarity in this contrast.

#### Exclusion

##### Not-my-turn (NMT) during Exclusion versus My-turn (MT) during Inclusion

There was an interaction effect between parent gender and brain activity for this contrast in a left-lateralized cluster containing IPS, PCC, SMA, precuneus, postCG, and preCG; a right-lateralized cluster covering extrastriate cortex, fusiform face area, IPS, middle occipital gyrus, cuneus, fusiform gyrus; as well as additional regions including left MCC, superior temporal gyrus (STG), superior and middle occipital gyri, as well as bilateral cerebellum (Table 5; Fig. 5B).

**Fig. 7:**
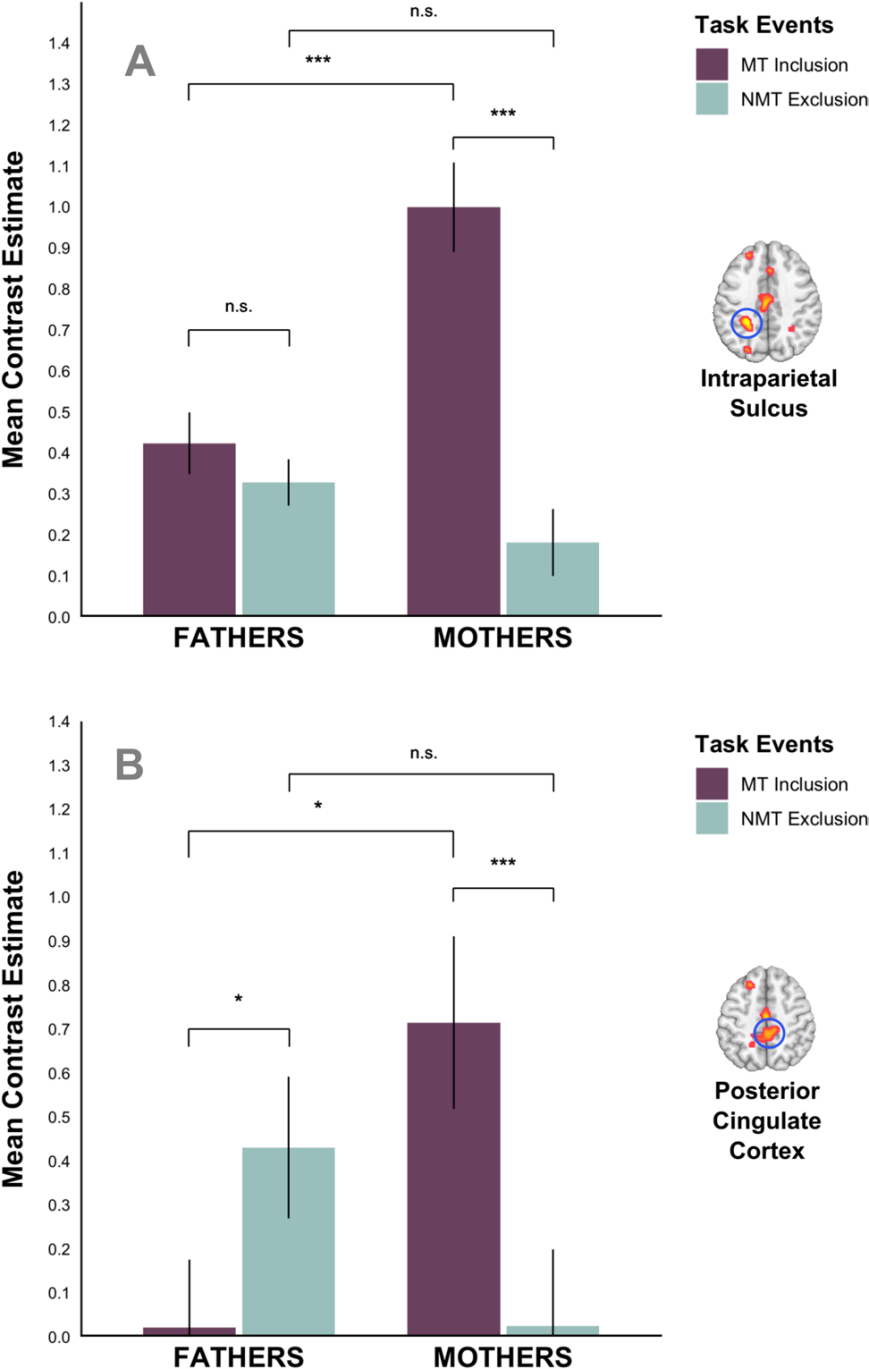
The bar plots display the mean activation (beta) values and their standard errors (y-axis) for the contrast, *NMT* during Exclusion (turquoise) > *MT* during Inclusion (purple) for mothers (*N* = 40) and fathers (*N* = 48; x-axis). Panel **A** illustrates the activity in a 5-mm–wide sphere within the left intraparietal sulcus (peak voxel at [−30 −40 36]). Panel **B** illustrates the activity in a 5-mm–wide sphere within the dorsal PCC (peak voxel at [0 −34 48]). n.s. = nonsignificant; *p < .05; ***p < .001

Again, we extracted beta values from several of the above regions (i.e., MCC, dorsal and ventral PCC, STG, left precuneus, bilateral IPS) to better characterize and illustrate this interaction. Two consistent patterns emerged within these ROIs (Fig. 7; see Supplementary Table S14 for detailed descriptive statistics): The first pattern was that mothers’ brain activity for MT during Inclusion was significantly higher (1) than that of fathers and (2) than mothers’ brain activity for NMT during Exclusion within the MCC (*F*_i_(1,86) = 15.88, *p*_i_ < .001; *F*_ii_(1,86) = 73.66, *p*_ii_ < .001), STG (*F*_i_(1,86) = 4.88, *p*_i_ = .03; *F*_ii_(1,86) = 14.63, *p*_ii_ < .001), IPS (*F*_i_(1,86) = 19.88, *p*_i_ < .001; *F*_ii_(1,86) = 70.07, *p*_ii_ < .001), dorsal PCC (*F*_i_(1,86) = 7.93, *p*_i_ = .006; *F*_ii_(1,86) = 10.74, *p*_ii_ = .002), and precuneus (*F*_i_(1,86) = 9,31, *p*_i_ = .003; *F*_ii_(1,86) = 27.11, *p*_ii_ < .001). A second emerging pattern was that (1) fathers’ brain activity was significantly higher for NMT during Exclusion than MT during Inclusion within the dorsal (*F*(1,86) = 4.53, *p* = .036) and ventral PCC (*F*(1,86) = 11.38, *p* = .001) and STG (*F*(1,86) = 5.67, *p* = .019), and that (2) fathers’ brain activity within ventral PCC for NMT during Exclusion was higher than that of mothers, *F*(1,86) = 4,69, *p* = .033. An additionally observed pattern was that fathers’ brain activity within MCC was significantly higher for MT during Inclusion than NMT during Exclusion, *F*(1,86) = 6,11, *p* = .015. We did not find any significant activation differences pertaining to familiarity in this contrast.

##### Not-my-turn (NMT) during Inclusion versus Exclusion

**Fig. 8:**
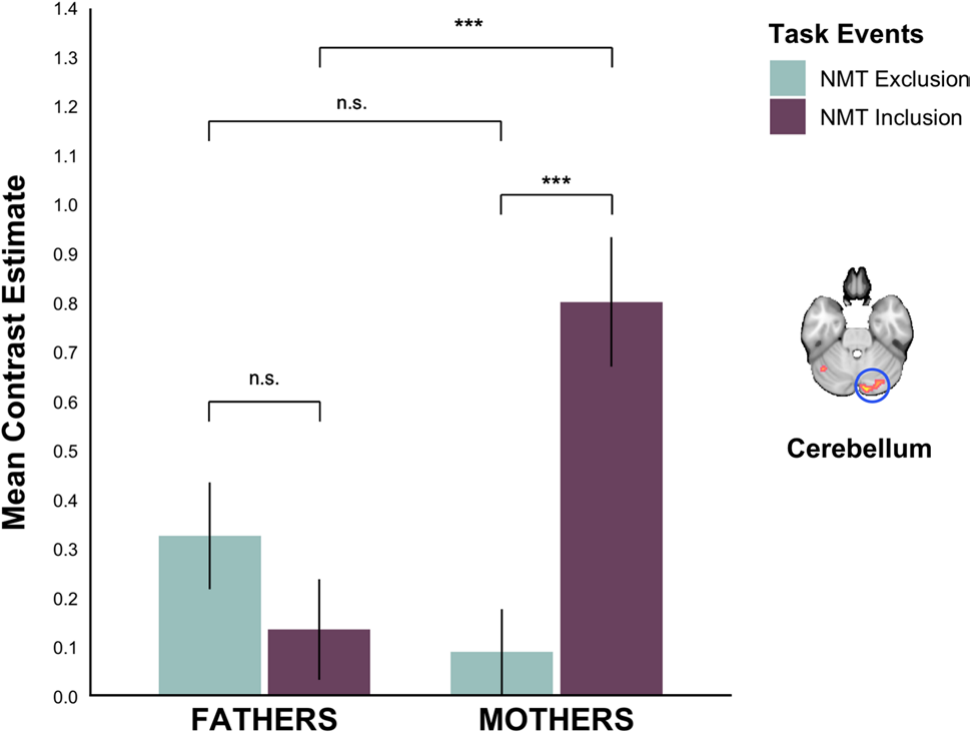
The bar plots display the mean activation (beta) values and their standard errors (y-axis) for the contrast *NMT* during Exclusion (turquoise) > *NMT* during Inclusion (purple) for mothers (*N* = 40) and fathers (*N* = 48; x-axis) in a cluster of the right cerebellum (peak voxel at [6 −82 −24], k = 267). n.s. = nonsignificant; *p < .05; ***p < .001

In this comparison, we observed a significant interaction between parent gender and brain activity in one cluster in the right cerebellum (peak voxel at [6 −82 −24], k = 267; Table 5; Fig. 5B). Further decomposition and illustration of this interaction through beta-extraction revealed that mothers’ response for NMT during Inclusion (*M* = .80, *SE* = .12) was significantly higher than fathers’ (*M* = .14, *SE* = .11), *F* = 16.428, *p* < .001 (Fig. 8). We did not find any significant activation differences pertaining to familiarity in this contrast.

### Child gender

As the last step, as it was preregistered, we tested whether parents’ neural responses differed as a function of their children’s gender, again by testing the eight main task contrasts and seven additional contrasts to test the effects of familiarity. No significant clusters emerged in any of these analyses.

### Post-hoc analyses of self-report measures

#### Parental involvement

##### Not-my-turn (NMT) during Exclusion versus My-turn (MT) during Inclusion

**Fig. 9:**
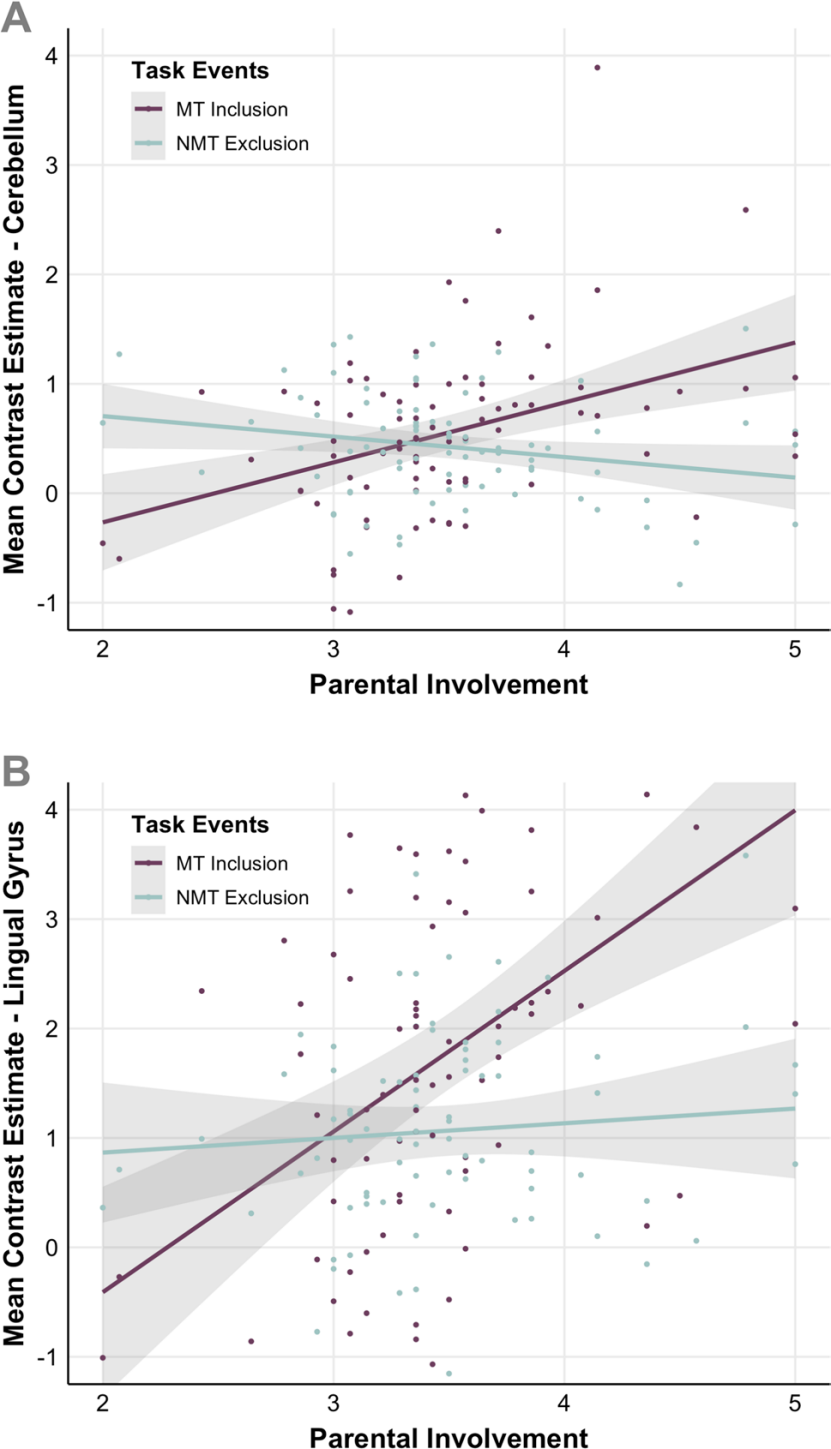
Scatter plots of the association between Parental Involvement (x-axis) and parents’ brain activity (y-axis) for MT during Inclusion (purple) and NMT during Exclusion (turquoise; *N* = 84). Panel **A** illustrates the association in a cerebellum cluster (peak voxel at [26 −70 −28], k = 1340), and Panel **B** in a lingual gyrus cluster (peak voxel at [−14 −74 2], k = 279)

Here, we only found an effect of Parental Involvement in two regions (see Supplementary Table S9): In a cerebellum cluster (peak voxel at [26 −70 −28], k = 1340) covering mainly the left Crus I and extending to bilateral Crus II, and a lingual gyrus cluster (peak voxel at [−14 −74 2], k = 279). Beta extraction for further examination and illustration revealed that the direction of the observed effect was similar in both regions, mainly being driven by a positive association between Parental Involvement and parents’ brain response for MT during Inclusion (Fig. 9). There were no significant interaction effects between Parental Involvement, parent gender, and the task events in these regions.

##### My-turn (MT) during Inclusion versus Reinclusion

**Fig. 10:**
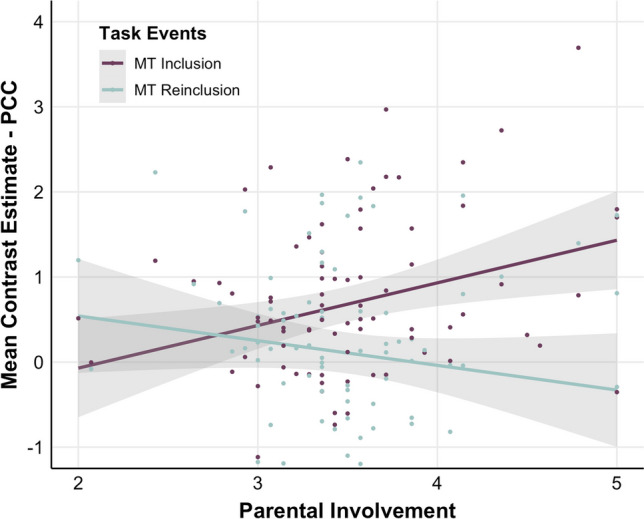
Scatter plot of the association between Parental Involvement (x-axis) and parents’ brain activity (y-axis) for MT during Inclusion (purple) versus Reinclusion (*turquoise; N* = 84) within a PCC cluster (peak voxel at [−4, −32, 48], k = 301)

For this contrast, we found an association between Parental Involvement and neural activity within the PCC, cuneus, and a left-lateralized cluster covering parts of the exterior cerebellum, lingual gyrus, and fusiform gyrus (Supplementary Table S10). The interaction patterns across these three clusters were identical, as illustrated by beta-extraction of activity in the PCC cluster (peak voxel at [−4, −32, 48], k = 301): Brain activity for MT was positively associated with Parental Involvement during Inclusion but negatively during Reinclusion (Fig. 10). There were no significant interaction effects between Parental Involvement, parent gender, and the task events in these regions.

##### Not-My-turn (NMT) during Inclusion versus Reinclusion

**Fig. 11:**
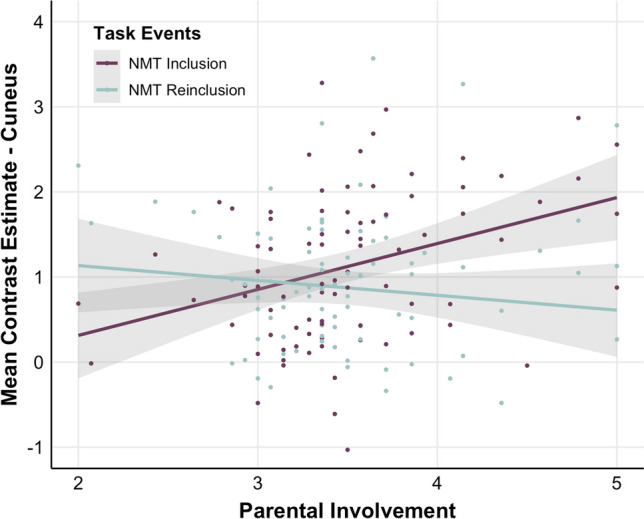
Scatter plot of the association between Parental Involvement (x-axis) and parents’ brain activity (y-axis) for NMT during Inclusion (purple) versus Reinclusion (turquoise; N = 84) within a cuneus cluster (peak voxel at [−8, −84, 14], k = 322)

There was a significant correlation between Parental Involvement and brain activity within the cuneus (Supplementary Table S11; Fig. 11). More precisely, beta-extraction and illustration of brain activity revealed that activity for NMT and Parental Involvement were positively associated during Inclusion but negatively during Reinclusion. There were no significant interaction effects between Parental Involvement, parent gender, and the task events in this region.

#### Delightful parenting

No significant effects of delightful parenting were observed in any of the contrasts tested.

#### Heightened Parenting

We observed an interaction effect between Heightened Parenting, brain activity during Inclusion and child familiarity (i.e., MT vs. NMT during Inclusion for own vs. unrelated child). This effect was observed within two large and several smaller clusters bilaterally (Supplementary Table S12). A large cluster within the right hemisphere covered the putamen, thalamus, aINS, pINS, STG, and inferior and medial frontal gyri (including dorsolateral prefrontal cortex). A second large cluster was present in the left hemisphere and covered the putamen, thalamus, STG, MTG, and basal ganglia. In addition, there were several significant smaller clusters in the TPJ, anterior prefrontal cortex (aPFC), preCG, and inferior occipital gyrus.

**Fig. 12:**
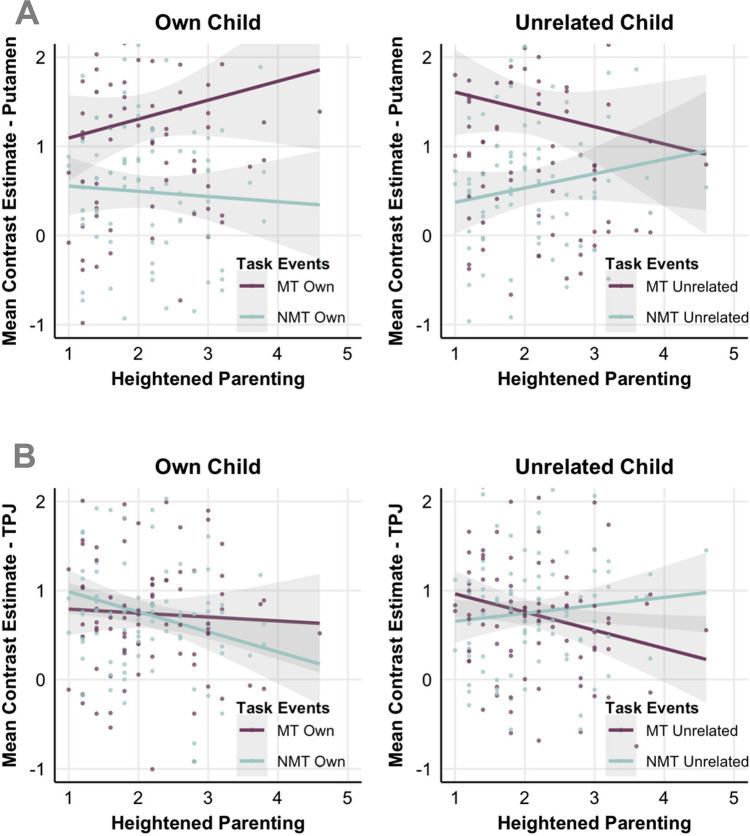
Scatter plots of the associations between Heightened Parenting (x-axis) and parents’ brain activity during Inclusion for MT and NMT events when playing with the Own versus Unrelated child (*N* = 86). Panel **A** illustrates activity in a 5-mm–wide sphere within Putamen (peak voxel at [−26 12 −2]). Panel **B** illustrates activity in a TPJ cluster (peak voxel at [52 −34 36], k = 634)

We subsequently extracted beta values from five representative regions (i.e., TPJ, aINS, dlPFC, aPFC, and dlPFC) to better understand and illustrate the exact nature of these complex interactions. This procedure revealed 1) one consistent pattern across the putamen, dlPFC, and aPFC, as illustrated by the 5-mm sphere in putamen (peak voxel at [−26 12 −2]; Fig. 12A), where, during the Inclusion phase, Heightened Parenting was positively associated with brain activity for MT and negatively for NMT when playing with the own child, but negatively associated with brain activity for MT and positively for NMT when playing with the unrelated child. 2) A second pattern that was observed in TPJ and aINS, as illustrated by the TPJ cluster (peak voxel at [52 −34 36], k = 634; Fig. 12B), was that, during the Inclusion phase, Heightened Parenting was negatively associated with brain activity for NMT when playing with the own child but positively for NMT when playing with the unrelated child. There were no significant interactions between Heightened Parenting, parent gender, task events, and child familiarity.

## Discussion

The nature of parent-offspring play and its contribution to child development is thought to vary, at least in part, as a function of parent gender (Paquette et al., 2020; Vallotton et al., 2020). We sought to compare, for the first time, the neural activity of mothers and fathers during a parent-child play episode: the virtual ball-tossing game Cyberball. Three main findings emerged: First, mothers and fathers alike activated neural circuits associated with the default-mode network (e.g., precuneus, AG), salience network (e.g., aINS, dACC), and emotion processing and regulation areas (e.g., vlPFC) during Exclusion versus Inclusion, broadly in keeping with previous Cyberball studies. Second, despite these comparable activation patterns, mothers and fathers responded to the phases of Cyberball with a differential pattern in some brain regions. During Inclusion, mothers activated areas subserving attention and reward processing more strongly compared with fathers. Conversely, during Exclusion, fathers more strongly activated areas subserving mentalizing compared with mothers. Third, we tested parents’ neural responses to Reinclusion in the game after Exclusion, finding a selective decrease in reward-related activity during this phase across both mothers and fathers. Finally while no activation differences emerged regarding child familiarity (e.g., own vs. unrelated child) and child gender in our main analyses, it proved relevant when Heightened Parenting was considered in the exploratory analyses. Here we observed a particular sensitivity of parents with heightened-anxious parenting style for events involving the own child, indicated by their elevated brain activity within mentalizing and saliency circuits. 

Parents’ overall neural activation pattern during Exclusion, covering the default-mode, salience, and emotion experience and regulation circuits, parallels recent work linking exclusion to activity within neural circuitries subserving social cognitive processes rather than “social pain” (Vijayakumar et al., 2017; Mwilambwe-Tshilobo & Spreng, 2021). Therefore, parents’ exclusion by children, including their own, evoked similar neural patterns as those found in adult Cyberball studies that involved exclusion by unfamiliar individuals. Furthermore, exploratory analyses including Parental Involvement revealed associations with parents’ neural response to inclusion and exclusion. Activity within the bilateral posterior cerebellum and left lingual gyrus during Inclusion, as opposed to Exclusion, correlated positively with Parental Involvement. Considering the posterior cerebellum’s role in social belief/sequence processing, especially during novel beliefs (Van Overwalle et al., 2020), this pattern of results suggests that more involved parents attributed more novelty to playing with children during Inclusion, possibly because they expected or preferred their child to interact with the other child. Besides, Parental Involvement was more positively associated with the neural response within the lingual gyrus during Inclusion as compared to Exclusion. Considering the role of the lingual gyrus in visual and tactile imagery (Olivetti Belardinelli et al., 2009), this result may suggest that involved parents recruited more resources for visual or tactile processing of the game (e.g., catching the ball) in a phase where they were included, suggesting that they were actually “more into the game.”

Interestingly, parent gender also played a role in shaping neural responses. Comparing mothers’ and fathers’ brain responses yielded more results indicating an absence—rather than the presence—of activation differences. In other words, the majority of conducted tests did not reveal any neural computation differences between mothers and fathers. This finding supports previous literature proposing that the paternal and maternal neurophysiology show substantial (albeit not a complete) overlap (Swain et al. 2014a; Feldman et al., 2019).

That said, our data also revealed some activation differences as a function of parent gender, which may mesh with theories postulating partly distinct roles for mothers and fathers as primary attachment and activation figures, respectively (Paquette et al., 2020; Feldman, 2023). In our study, mothers, while receiving the ball during the Cyberball Inclusion phase (*my-turn* [MT] > *not-my-turn* [NMT]), recruited circuits subserving attention (e.g., IPS), social cognition (e.g., PCC/precuneus), and reward processing (e.g., putamen) more strongly compared with fathers. Mothers’ stronger engagement of reward- and attention-related networks within an approach scenario (i.e., receiving the ball) concords with the behavioral literature illustrating that mothers are more responsive in a play situation with their infants and preschool-aged children (Vallotton et al., 2020). The most recent neurophysiological models of parenting highlight the primacy of reward, saliency, and motivation networks in mothering as the central hubs of a “parenting network” (Feldman et al., 2019; Abraham & Feldman, 2022). From this perspective, mothers’ neural response during Inclusion also could be framed as an increased salience of child cues with the aim of attunement and bond formation (Swain et al., 2014b).

Conversely, fathers (compared with mothers) showed stronger activation for Exclusion (NMT during Exclusion > MT during Inclusion) mainly in areas associated with mentalizing (i.e., PCC, STG). This mentalizing response might be attributable to a key aspect of their proposed activation role, namely, that of mediating the child’s relationship with the external world (Paquette & St. George, 2023). Parents’ exclusion in Cyberball means their children’s exclusive play interaction with another child, i.e., an agent external to the family bond. Thus, fathers’ increased mentalizing activity during play between the two children may reflect their attempt to assess “external contextual factors” and monitor the safety of their child (Swain et al., 2014a). Alternatively, fathers’ mentalizing activity during exclusion might imply the involvement of a social monitoring system (Pickett & Gardner, 2005), which is thought to optimize the odds of reconnection after exclusion by enhancing sensitivity to social cues. This is a known reaction following exclusion (Molden & Maner, 2013), which may also facilitate fathers taking on the role of the “primary activation” figure (Paquette & St. George, 2023) that challenges children and therefore seeks reinclusion (von Klitzing & White, 2020). Finally, parent gender differences in Cyberball align with two previous studies that compared mothers’ and fathers’ response to own infant stimuli (Abraham et al., 2014; Atzil et al., 2012), finding stronger limbic/subcortical activations in mothers and stronger cortical/social-cognitive activations in fathers.

Our interpretation notably begs the question whether activation differences for mothers and fathers might be more readily attributable to biological sex, per se. Our design cannot conclusively tease apart these competing interpretations. Most previous literature suggests that females, relative to males, attend more closely to social cues (Su et al., 2009), which is evidenced by their physiological reactivity (Benenson et al., 2013) and amygdala-frontal network activity (Bürger et al., 2023) following exclusion. Accordingly, mothers’ elevated sensitivity to Inclusion in our study aligns with these gender-dependent patterns. However, fathers’ elevated neural response to Exclusion clearly diverges from the diminished responsiveness to exclusion typically detected for males relative to females in previous work. Therefore, gender-specific parent roles appear to be a better account for this activation pattern than biological sex, per se.

Next, we tested parents’ neural responses to Reinclusion in the game after Exclusion. There was an overall decrease in neural activity for Reinclusion relative to Inclusion, which could be explained by habituation. However, besides this, we also observed a selective decrease mainly in reward circuits (e.g., putamen) as a response to MT versus NMT during Reinclusion compared with Inclusion. It was previously shown that the effects of exclusion may persist up to 45–55 min (Buelow et al., 2015). Thus, the selective decrease in reward activity seems to be a carry-over effect of the exclusion experience for parents. Previous literature suggests that neural response to reinclusion may index the individual’s particular response to a reconnection scenario following exclusion (Maurage et al., 2012; White et al., 2013; Heeren et al., 2017). Hence, reentering the ballgame after the exclusion episode was likely less rewarding for parents compared with playing with them at the beginning of the game. This effect was especially pronounced for more involved parents. Our results concerning Parental Involvement thus suggest that involved parents were more engaged with the game during Inclusion and, conversely, less so during Reinclusion, as evidenced by activation patterns within the PCC, cuneus, and cerebellum, amongst others. From the perspective of Cyberball game dynamics, involved parents seemed to make themselves available for children from the get-go and, crucially, at the same time became *less threatened* by Exclusion, as evidenced by their responses during Reinclusion. Conversely, the tables seemed to turn for less involved parents, such that they seemed to become more responsive during Reinclusion, only after Exclusion, which possibly threatened their inclusionary status more strongly, given their lower levels of overall involvement as a parent. Activation in neural circuits subserving attention and social cognition may therefore facilitate social monitoring to enhance parents’ efforts to reconnect with their children (Molden & Maner, 2013; Pickett & Gardner, 2005). Finally, we found no significant differences between mothers and fathers in their responses during the Reinclusion phase.

Lastly, we also examined whether parents had distinct neural responses to own versus unrelated child during Cyberball. Here, while whole brain contrasts revealed no differences between child familiarity conditions, the only significant difference emerged when Heightened Parenting was considered in the analyses. Heightened Parenting reflects a parenting style that discourages autonomy and seeks excessive proximity (Røhder et al., 2019). Heightened-anxious parenting was associated with parents’ neural activity within multiple brain regions during Inclusion as a response to NMT versus MT events when playing with the own versus unrelated child. Specifically, heightened-anxious parenting was positively associated with activity within the putamen, dlPFC, and aPFC/vACC when the own child received the ball from the unrelated child, and when parents received the ball from their own child. Conversely, it was negatively associated with brain activity when the unrelated child either got the ball or threw it to their parent. Accordingly, the pattern of activity within reward and emotion processing circuits suggests a preference to play exclusively with the own child as the parent has a more pronounced anxious parenting style. Besides, heightened-anxious parenting was also positively associated with activity within the TPJ and aINS especially when the own child received the ball from the unrelated child and negatively associated with activity within these regions when the unrelated child received the ball from the own child. This pattern suggests that parents with a more pronounced anxious parenting style attributed more saliency to and mentalized more during their own child’s ball-play with unfamiliar children, potentially reflecting an excessive need to attend to and monitor their child’s contact attempts with others. This may resemble what is observed in the anxious attachment style (Long et al., 2020). Taken together, a heightened-anxious parenting style seemed to be associated with an increased need to interact with the own child and particular attentiveness to the cues pertaining to the own offspring as they ventured beyond the safety of the parent-child dyad.

It is noteworthy that our whole brain contrasts did not reveal any other significant differences pertaining to the comparison between the own and the unrelated child, contrary to our expectations. This may initially seem surprising, given that much of the parenting literature builds on similar contrasts. One possible explanation for this null finding is methodological. To our knowledge, ours was the first fMRI study to compare brain activity associated with two different Cyberball coplayers. Although such a contrast has been used in ERP studies (Sreekrishnan et al., 2014), the fMRI signal might not possess sufficient signal-to-noise ratio to detect effects with about 15 events. Indeed, in such a fast-paced dynamic paradigm, the lower temporal resolution of fMRI alongside the delayed hemodynamic response may place a higher upper bound on distinguishing events that lie in such close temporal proximity to one another. We therefore suggest future studies to build on this fMRI contrast by including more events in their design, as dissecting coplayer specific activity in Cyberball might bear fruit for understanding complex interaction dynamics.

A few limitations of this study deserve consideration. First, as noted above, our design cannot fully rule out competing interpretations, especially about neural differences attributable to parent gender. While it is virtually impossible to resolve this issue within a Cyberball design like ours without changing its fundamental aim and nature, future studies may consider including additional nonchild coplayer and unrelated but familiar child conditions that may help isolate additional effects of interest. Second, as suggested above, the design could be further improved by including more events in the task, especially for comparisons of activity associated regarding the two coplayers (the trade-off is a lengthier paradigm). Turning to the strengths, ours is the first study to recruit large samples of both mothers and fathers to study parent-child play in an interactive context. We believe that research aiming to establish neural correlates of parenting should capitalize on such interaction paradigms more often, given their increased ecological validity compared with simple stimulus-presentation tasks.

### Conclusions

Collectively, our data on neural responses during Cyberball lead us to surmise that playing with one’s own and an unfamiliar preschool-aged child typically engages comparable neural networks across both mothers and fathers. However, at the same time, we also detected mother- and father-specific neural activation that may highlight distinct priorities of their neurobiological systems: sensitivity and responsiveness in mothers, underpinned by attention and reward-related neural circuits during Inclusion, versus social-monitoring in fathers, underpinned by neural circuits subserving mentalizing. These observed differences between mothers and fathers are seemingly due to a combined influence of biological and social factors. Indeed, both of these factors have parenting-independent and parenting-related components. Therefore, it is crucial to be aware of this complexity and to approach our results with appropriate caution.

A possible next step could be to consider maternal and paternal contributions within a single system, because the father-child relationship is not only a relationship in its own right but also transforms the mother-child relationship or vice versa (Belsky, 1981). Therefore, it might bear fruit to develop paradigms to study triadic dynamics within the known methodological limitations by either using combined single-subject designs or hyperscanning.

## Supplementary information

Below is the link to the electronic supplementary material.

Supplementary file1 (PDF 642 KB)

Supplementary file2 (PDF 112 KB)

## Data Availability

The data, materials, and code that support the findings of this study cannot be made publicly available due to data protection laws. However, they will be made available upon reasonable request and after signing a data-sharing agreement with the corresponding author PV. This study was preregistered.
