# Supplementary material for: Caregiver or Playmate? Fathers’ and mothers’ brain responses to ball-play with children
Source: Cogn Affect Behav Neurosci. 2024 Dec 5;25(2):434–53. doi: 10.3758/s13415-024-01237-1 (PMC11906569; doi:10.3758/s13415-024-01237-1)
Supplement: Supplementary file 1 — Supplementary file1 (PDF 642 KB) [file 13415_2024_1237_MOESM1_ESM.pdf]

Caregiver or Playmate? Fathers' and mothers' brain responses to ball-play with children

SUPPLEMENTARY MATERIALS

Methods

Sample

This fMRI study was part of a larger project assessing the neurobiology of caregiver-child relationships in a total sample of *N*=147 caregivers with their 5 to 6-year-old biologically related children. All participants were pre-screened for medical, neurological, and psychological/psychiatric issues, substance abuse, handedness, reading and writing difficulties, as well as fMRI counter-indications.

The original aim was to have *N*=100 good-quality fMRI data sets from mothers and fathers. Due to many parents meeting fMRI exclusion criteria and additional dropouts before fMRI scanning, parents were over-recruited for the remaining parts of the project. Eventually, *N*=50 fathers were eligible to participate in the present study. Data acquisition had to be halted prematurely in mothers due to the COVID-19 pandemic so that only *N*=41 fMRI scans could be obtained from mothers.

Results: Whole-brain Effects of Task Conditions

Hereby, we report the results from the pre-registered whole-brain analyses that were not mentioned in the main text. We did not find any significant activation differences pertaining to familiarity in neither of the following contrasts. All the reported results below combined the familiarity conditions, testing parents' neural response during play with both children.

Inclusion

a. My-turn (MT) > Not-my-turn (NMT) within Inclusion

MT as compared to NMT events during Inclusion revealed stronger activation within the central-executive network (i.e., lateral frontoparietal control) , including bilateral dorsolateral prefrontal cortex (dlPFC), precuneus, and thalamus; the saliency network, including the bilateral anterior insula (aINS), dorsal anterior cingulate cortex (dACC); the somatomotor circuits including the bilateral premotor cortex, supplementary motor area (SMA), precentral (preCG) and postcentral (postCG) gyri; visual-processing areas, including the bilateral extrastriate cortex and lingual gyrus; as well as additional areas, such as bilateral putamen, superior parietal lobule, and cerebellum (see Table S1 and Figure S1).

b. My-turn (MT) during Inclusion > Not-my-turn (NMT) during exclusion

MT during Inclusion compared to NMT during Exclusion activated the central-executive network, including the bilateral dlPFC, precuneus, and thalamus; the saliency network (e.g., bilateral dACC, aINS); and the reward circuitry (e.g., bilateral putamen). Additional areas that showed increased activity were bilateral somatomotor cortices, middle occipital gyrus, lingual gyrus, cerebellum (see Table S2 and Figure S2).

Not-my-turn events

a. Not-my-turn (NMT) during Inclusion > Exclusion

Whole-brain analyses showed heightened neural activity for NMT during Inclusion relative to Exclusion in the nodes of the salience network, including aINS, dACC, dlPFC, thalamus, intraparietal lobule (IPL), and hypothalamus. Additional regions that showed heightened activity

were the postCG, SMA, superior (SFG) and medial frontal gyri (MFG), precuneus, putamen, and cerebellum (see Table S3 and Figure S3).

b. Not-my-turn (NMT) during Inclusion versus Re-inclusion

NMT during Inclusion relative to Re-inclusion activated the areas associated with the default mode network, including the bilateral ventromedial prefrontal cortex (vmPFC), posterior cingulate cortex (PCC), middle temporal gyrus (MTG), superior temporal gyrus (STG), supramarginal gyrus, parahippocampal gyrus, right precuneus, and right temporoparietal junction (TPJ); the salience network including bilateral aINS, ACC, and inferior frontal gyrus (IFG); visual-processing, including the bilateral striate and extrastriate cortices, cuneus, fusiform gyrus, and right lingual gyrus; somatomotor related activity, including the premotor cortex and SMA; as well as additional regions including bilateral pons/midbrain, parahippocampal gyrus/amygdala, left putamen, and right temporal pole (see Table S4 and Figure S4). The inverse contrast did not yield any significant clusters.

Re-inclusion

a. MT versus NMT within Re-inclusion

NMT as compared to MT events during Re-inclusion activated the default mode network nodes including bilateral medial prefrontal cortex (mPFC; as two separate clusters in dorsal and orbitofrontal areas), PCC, angular gyrus (AG), and MTG; and somatomotor network including bilateral preCG, postCG, and SMA. Other regions that showed increased activity include bilateral rostral ACC, ventral ACC, posterior insula (pINS), dlPFC, ventrolateral prefrontal cortex (vlPFC) and left dACC (see Table S5 and Figure S5).

The reverse contrast (MT > NMT) revealed activation in the salience network including the bilateral dACC, aINS, thalamus, and dlPFC, as well as the somatomotor network including the bilateral SMA, premotor cortex, preCG, and left postCG. Other regions that showed increased activity were the bilateral middle occipital gyrus, precuneus, lingual gyrus, midbrain (red nucleus), and cerebellum (see Table S6 and Figure S6).

b. My-Turn (MT) during Inclusion versus Re-inclusion

Whole-brain level comparison of MT during Inclusion relative to Re-inclusion revealed activity in visual-processing areas, including bilateral superior occipital gyrus, cuneus, superior parietal lobule, lingual gyrus, as well as somatomotor network including bilateral preCG and postCG, extending towards SFG; reward-related circuits (e.g. bilateral putamen), and additional areas such as bilateral mid-anterior cingulate cortex (ACC), cerebellum, IFG, STG, and left temporal pole (see Table S7 and Figure S7). The inverse contrast did not yield any significant clusters.

c. Throw during Inclusion versus Re-inclusion

Comparison of throw events during Inclusion compared to Re-inclusion revealed significant activation in the somatomotor network (e.g., left preCG, bilateral postCG, and SMA); visual-processing (e.g., bilateral cuneus, extrastriate cortex, fusiform gyrus, and lingual gyrus); and default-mode network nodes including bilateral PCC, vmPFC, IPL, amygdala, and left IFG. Other regions that showed increased activity in this contrast included bilateral dACC, parahippocampal gyrus, STG, cerebellum, and right hippocampus (see Table S8 and Figure S8). The inverse contrast did not yield any significant clusters.

Supplementary Figures

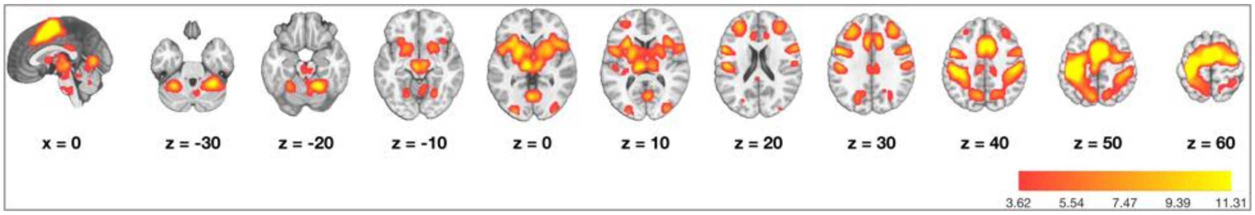

Supplementary Figure S1. Whole brain activation differences (*N*=88) for the contrast, *MT* > *NMT* during Inclusion. Random-effects (one-sample t-test), $p < .001$ uncorrected at the voxel level and $p < .05$ FWE-corrected at the cluster level.

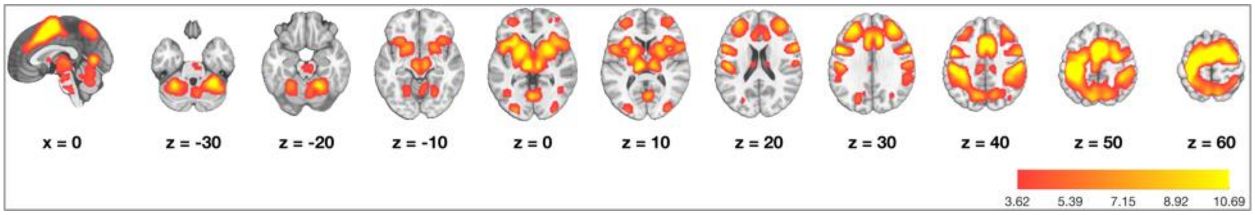

Supplementary Figure S2. Whole brain activation differences (*N*=88) for the contrast *MT* during Inclusion > *NMT* during Exclusion. Random-effects (one-sample t-test), *p*< .001 uncorrected at the voxel level and *p*< .05 FWE-corrected at the cluster level.

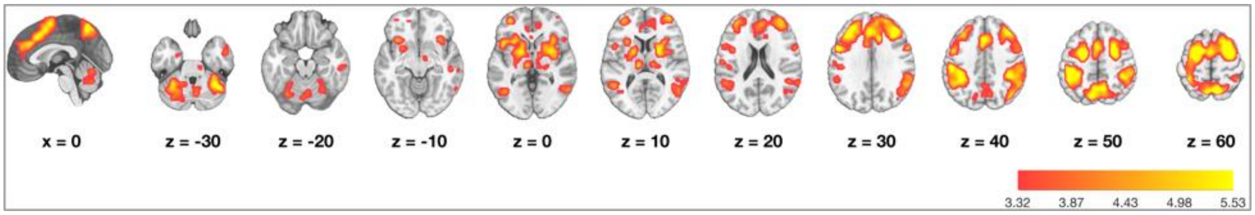

Supplementary Figure S3. Whole brain activation differences (*N*=88) for the contrast *NMT* during Inclusion > *NMT* during Exclusion. Random-effects (one-sample t-test), $p < .001$ uncorrected at the voxel level and $p < .05$ FWE-corrected at the cluster level.

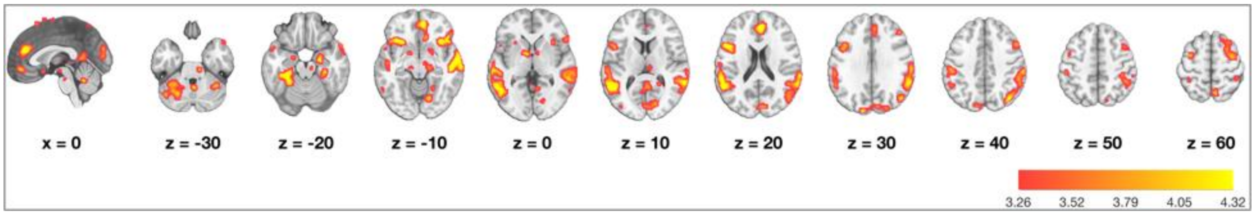

Supplementary Figure S4. Whole brain activation differences (*N*=88) for the contrast *NMT* during Inclusion > *NMT* during Reinclusion. Random-effects (one-sample t-test), $p < .001$ uncorrected at the voxel level and $p < .05$ FWE-corrected at the cluster level.

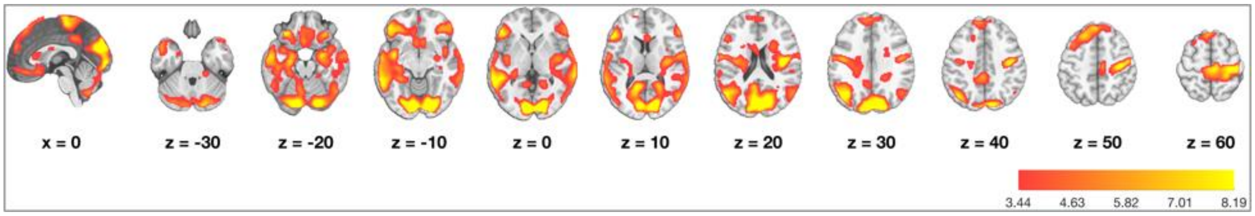

Supplementary Figure S5. Whole brain activation differences (*N*=88) for the contrast *NMT* > *MT* during Reinclusion. Random-effects (one-sample t-test), *p*< .001 uncorrected at the voxel level and *p*< .05 FWE-corrected at the cluster level.

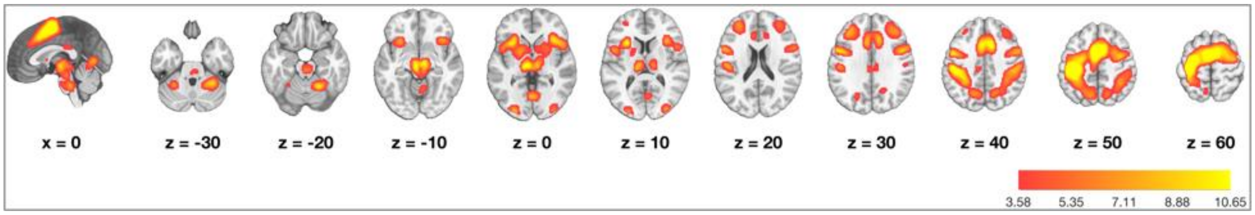

Supplementary Figure S6. Whole brain activation differences (*N*=88) for the contrast *MT* > *NMT* during Reinclusion. Random-effects (one-sample t-test), $p < .001$ uncorrected at the voxel level and $p < .05$ FWE-corrected at the cluster level.

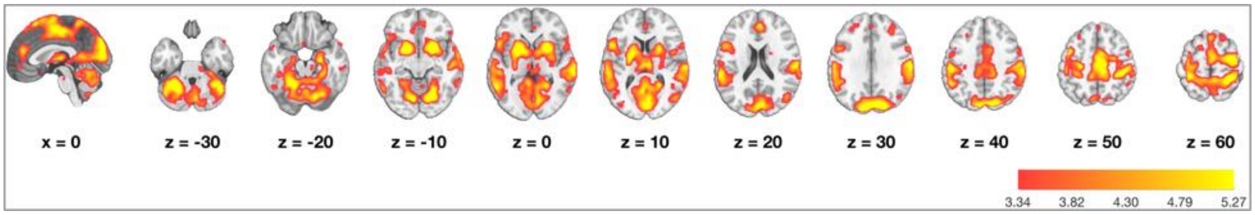

Supplementary Figure S7. Whole brain activation differences (*N*=88) for the contrast *MT* during Inclusion > *MT* during Reinclusion. Random-effects (one-sample t-test), $p < .001$ uncorrected at the voxel level and $p < .05$ FWE-corrected at the cluster level.

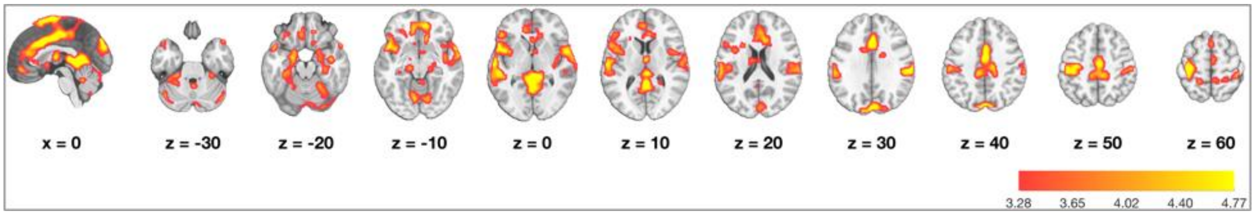

Supplementary Figure S8. Whole brain activation differences (*N*=88) for the contrast *Throw* during Inclusion > *Throw* during Reinclusion. Random-effects (one-sample t-test), $p< .001$ uncorrected at the voxel level and $p< .05$ FWE-corrected at the cluster level.

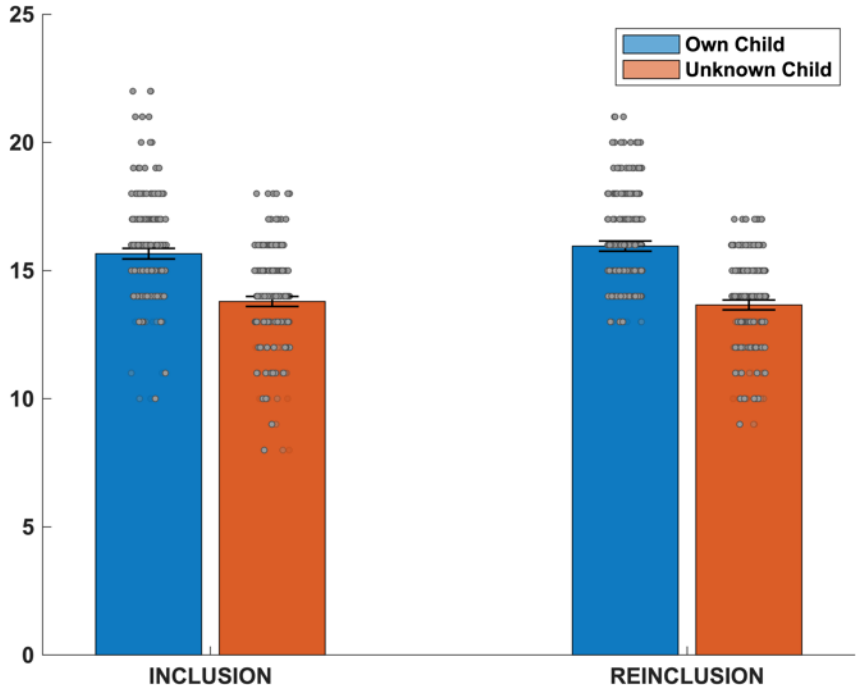

Supplementary Figure S9. Bar plot illustrating parents' throw choices (i.e., number of throws; y-axis) to their Own child (blue) and the Unknown child (red) during Inclusion and Re-inclusion (x-axis).

Supplementary Tables

Table S1

Whole brain activations for my-turn (MT) > not-my-turn (NMT) during Inclusion (N=88)

Anatomical Region	Hemisphere	x,y,z	*t*	*z*	*k*	*p*
Precentral Gyrus	L	-38, -22, 56	26,97	Inf	28028	<.001
SMA	L	-6, -4, 58	22,36	Inf		
Premotor cortex	L	-30, -14, 66	19,12	Inf		
SMA	R	6, 2, 54	18,87	Inf		
Premotor cortex	R	36, -10, 56	13,9	Inf		
dACC	BL	8, 16, 40	13,36	Inf		
Thalamus	L	-10, -18, 2	13,04	Inf		
Premotor cortex/ IFG	L	-50, 2, 36	12,81	Inf		
Anterior insula	L	-30, 18, 8	12,44	Inf		
Putamen	L	-24, 0, 4	11,5	Inf		
Cerebellum	R	18, -50, -20	15,09	Inf	2668	<.001
Lingual gyrus	BL	-4, -68, 4	10,94	Inf		
Postcentral gyrus	R	44, -26, 42	11,73	Inf	3329	<.001
Precuneus	R	14, -66, 44	7,04	6,25		
Superior parietal lobule	R	28, -56, 50	4,16	3,96		
Cerebellum	L	-30, -52, -26	10,86	Inf	1007	<.001
Lingual gyrus	L	-22, -66, -6	5,33	4,94		
dlPFC	L	-32, 36, 28	10,19	Inf	1345	<.001

dlPFC	R	32, 38, 28	9,76	Inf	1197	<.001
Cerebellum	R	16, -60, -50	9,3	7,72	491	0,003
Middle occipital gyrus	R	32, -88, 8	8,58	7,28	345	0,014
Middle occipital gyrus	L	-28, -92, 4	8,53	7,25	265	0,039

Note. Clusters listed are significant in a whole-brain analysis ($p<.001$ uncorrected at voxel level and $p < 0.05$ FWE-corrected at cluster level, $k > 20$ voxels). x, y, z refer to MNI coordinates. T refers to the t-score and z the z-score at those coordinates (local maxima). K refers to the number of voxels in each significant cluster. BL refers to bilateral.

Table S2

Whole brain activations for my-turn (MT) during Inclusion > not-my-turn (NMT) during Exclusion (N=88)

Anatomical Region	Hemisphere	x,y,z	*t*	*z*	*k*	*p*
Precentral Gyrus	L	-38, -22, 56	21,1	Inf	42944	<.001
SMA	BL	-4, -4, 60	18,84	Inf		
Thalamus	L	-10, -20, 4	14,52	Inf		
Premotor cortex	L	-56, 6, 28	14,15	Inf		
dACC	R	10, 20, 32	13,54	Inf		
Anterior insula	L	-30, 16, 6	13,46	Inf		
Premotor cortex	R	24, -4, 62	13,35	Inf		
Putamen	L	-22, 2, 4	12,76	Inf		
dlPFC	R	32, 38, 32	12,18	Inf		
dlPFC	L	-30, 38, 32	11,78	Inf		
Thalamus	R	8, -18, 2	11,69	Inf		
Cerebellum	R	34, -48, -30	12,59	Inf	6197	<.001
Cerebellum	L	-32, -50, -28	10,59	Inf		
Lingual gyrus	BL	-4, -68, 4	10,32	Inf		
Cerebellum	R	24, -56, -54	9,74	Inf	735	<.001
Middle occipital gyrus	L	-28, -90, 2	7,87	6,82	335	0,016
Middle occipital gyrus	R	32, -88, 6	6,57	5,9	303	0,024

Note. Clusters listed are significant in a whole-brain analysis (p<.001 uncorrected at voxel level and p < 0.05 FWE-corrected at cluster level, k > 20 voxels). x, y, z refer to MNI coordinates. T refers to the t-score and z the z-

score at those coordinates (local maxima). K refers to the number of voxels in each significant cluster. BL refers to bilateral.

Table S3

Whole brain activations for not-my-turn (NMT) during Inclusion > not-my-turn (NMT) during Exclusion (N=88)

Anatomical Region	Hemisphere	x,y,z	*t*	*z*	*k*	*p*
SFG	R	22, 6, 58	8,59	7,29	27132	<.001
MFG	L	-24, -6, 54	8,03	6,93		
MFG	R	24, 4, 48	7,58	6,62		
Precuneus	R	6, -62, 58	7,52	6,58		
Postcentral gyrus	L	-54, -24, 38	6,65	5,96		
SMA	R	16, 18, 64	6,6	5,93		
Inferior parietal lobule	R	36, -36, 44	6,54	5,88		
Precuneus	L	-10, -66, 54	6,41	5,79		
Anterior insula	L	-30, 16, 6	6,16	5,59		
dACC	BL	-4, 22, 38	6,1	5,55		
Cerebellum	R	26 -54 -52	6,42	5,79	382	<.001
Cerebellum	R	36, -50, -32	7,58	6,62	994	<.001
Putamen	R	20, 0, 8	6,34	5,73	1851	<.001
Anterior insula	R	32, 22, -4	5,07	4,74		
Thalamus (Medial dorsal nucleus)	R	10, -20, 6	5,07	4,73		
Hypothalamus	R	10, -6, -4	4,1	3,91		
Cerebellum	L	-34, -50, -30	5,67	5,21	2544	<.001
Cerebellum	BL	4, -66, -30	4,43	4,2		

Note. Clusters listed are significant in a whole-brain analysis ($p<.001$ uncorrected at voxel level and $p < 0.05$ FWE-corrected at cluster level, $k > 20$ voxels). x, y, z refer to MNI coordinates. T refers to the t-score and z the z-score at those coordinates (local maxima). K refers to the number of voxels in each significant cluster. BL refers to bilateral.

Table S4

Whole brain activations for not-my-turn (NMT) during Inclusion > not-my-turn (NMT) during Reinclusion (N=88)

Anatomical Region	Hemisphere	x,y,z	*t*	*z*	*k*	*p*
STG	L	-52, -54, 12	5,89	5,39	2645	<.001
MTG	L	-50, -58, 2	4,39	4,16		
Postcentral gyrus	L	-56, -24, 46	4,15	3,95		
Supramarginal gyrus	L	-62, -28, 32	3,9	3,73		
MTG	R	50, -22, -6	5,82	5,33	4160	<.001
STG	R	62, -46, 14	5,08	4,74		
Precuneus	R	36, -74, 40	5,05	4,71		
Supramarginal gyrus	R	62, -36, 24	4,65	4,38		
Temporal pole	R	48, 14, -36	4,42	4,18		
TPJ	R	58, -44, 30	4,4	4,17		
Postcentral gyrus	R	62, -26, 38	4,24	4,03		
Inferior parietal lobule/Angular gyrus	R	52, -38, 38	4,02	3,84		
IFG/Anterior insula	R	32, 20, -4	5,62	5,18	554	<.001
IFG/ Anterior insula	L	36, 22, -10	5,47	5,05	380	<.001
STG	L	54, 10, -12	4,4	4,17		
ACC	BL	4, 42, 20	5,25	4,88	450	<.001
Parahippocampal gyrus	L	30, -32, -20	5,13	4,78	1089	<.001
Fusiform gyrus	L	-40, -34, -20	4,59	4,33		

Cerebellum	L	-26, -62, -28	4,15	3,95		
IFG	L	-50, 12, 20	5	4,68	670	<.001
Parahippocampal gyrus / Amygdala	R	20, -10, -16	4,89	4,59	492	<.001
Pons / Midbrain	R	8, -24, -24	4,55	4,3		
Putamen	L	-10, 0, 0	4,88	4,57	277	<.001
Parahippocampal gyrus / Amygdala	L	-16, 0, -12	4,13	3,93		
Pons / Midbrain	L	-14, -20, -10	3,6	3,47		
Cerebellum	L	-14, -56, -50	4,85	4,55	263	<.001
SMA	L	-18, 4, 72	4,8	4,51	426	<.001
Premotor cortex	R	-36, -2, 60	4,21	4		
vmPFC	R	4, 48, -8	4,63	4,37	412	<.001
Extrastriate cortex	L	-16, -92, 28	4,47	4,23	1050	<.001
Lingual gyrus	R	14, -72, -6	4,24	4,03		
Striate cortex	R	16, -80, 8	4	3,82		
Extrastriate cortex/Cuneus	BL	6, -84, 18	3,92	3,75		
SMA/Premotor cortex	R	30, -2, 64	4,4	4,17	359	<.001
PCC	BL	2, -52, 10	3,84	3,68	264	<.001

Note. Clusters listed are significant in a whole-brain analysis ($p<.001$ uncorrected at voxel level and $p < 0.05$ FWE-corrected at cluster level, $k > 20$ voxels). x, y, z refer to MNI coordinates. T refers to the t-score and z the z-score at those coordinates (local maxima). K refers to the number of voxels in each significant cluster. BL refers to bilateral.

Table S5

Whole brain activations for not-my-turn (NMT) > my-turn (MT) during Reinclusion (N=88)

Anatomical Region	Hemisphere	x,y,z	*t*	*z*	*k*	*p*
Lingual gyrus	R	14, -84, -10	18,91	Inf	37232	<.001
Lingual gyrus	L	-10, -90, -8	16,89	Inf		<.001
Cuneus	R	8, -88, 30	13,51	Inf		<.001
Cuneus	L	-8, -86, 30	12,28	Inf		<.001
Posterior insula	R	38, -14, 20	12,08	Inf		<.001
Precentral gyrus	R	32, -26, 58	10,51	Inf		<.001
STG	L	-62, -44, 4	10,37	Inf		<.001
Angular gyrus	L	-46, -70, 32	10,36	Inf		<.001
PCC	L	-14, -56, 12	8,88	7,47		<.001
Postcentral gyrus	BL	-4, -26, 64	8,64	7,32		<.001
dlPFC	L	-54, 28, 12	10,16	Inf	4341	<.001
vlPFC	L	-32, 34, -16	9,51	7,84		<.001
vlPFC	R	32, 34, -14	8,52	7,24		<.001
Orbitofrontal cortex (OFC)	R	36, 38, -12	8,42	7,18		<.001
Subcallosal gyrus/subgenual ACC	BL	-6, 18, -14	7,45	6,53		<.001
dlPFC	R	50, 44, 0	6,05	5,51		<.001
OFC	L	-20, 22, -14	5,54	5,11		<.001
dmPFC	L	-6, 40, 52	7,89	6,83	2472	<.001

SFG	L	-22, 26, 48	7,54	6,6		<.001
SMA	L	-6, 20, 68	7,15	6,32		<.001
SFG	R	4, 42, 52	5,5	5,08		<.001
MFG	L	-42, 16, 50	5,48	5,07		<.001
dmPFC	R	6, 54, 36	4,26	4,05		<.001
Ventral (pregenual) anterior cingulate cortex (vACC)	R	4, 22, 10	4,31	4,1	242	<.001
dACC	L	-6, 4, 22	4,2	4		<.001

Note. Clusters listed are significant in a whole-brain analysis ($p<.001$ uncorrected at voxel level and $p < 0.05$ FWE-corrected at cluster level, $k > 20$ voxels). x, y, z refer to MNI coordinates. T refers to the t-score and z the z-score at those coordinates (local maxima). K refers to the number of voxels in each significant cluster. BL refers to bilateral.

Table S6

Whole brain activations for my-turn (MT) > not-my-turn (NMT) during Reinclusion (N=88)

Anatomical Region	Hemisphere	x,y,z	*t*	*z*	*k*	*p*
Precentral Gyrus	L	-38, -20, 54	21,31	Inf	22317	<.001
SMA	BL	-4, -6, 56	18,38	Inf		
Premotor cortex	L	-32, -12, 58	14,54	Inf		
dACC/SMA	R	8, 8, 48	14,34	Inf		
Thalamus	L	-6, -16, -2	12,89	Inf		
Midbrain (red nucleus)	R	6, -16, -4	12,71	Inf		
Midbrain (red nucleus)	L	-6, -24, -6	12,31	Inf		
Anterior insula	L	-30, 18, 8	12,08	Inf		
Anterior insula	R	32, 22, 6	10,66	Inf		
Premotor cortex	R	48, 4, 34	10,06	Inf		
Cerebellum	R	22, -52, -22	11,8	Inf	1419	<.001
Lingual gyrus	BL	-2, -68, 2	8,11	6,98		
Middle occipital gyrus	L	-30, -92, 4	8,71	7,36	279	0,030
Intraparietal sulcus	R	46, -28, 42	8,34	7,13	2508	<.001
Precuneus	R	16, -64, 44	7,11	6,29		
dlPFC	L	-34, 38, 28	7,79	6,76	1057	<.001
Middle occipital gyrus	R	32, -88, 8	7,4	6,5	267	0,035
Cerebellum	L	-30, -50, -28	7,15	6,32	266	0,036
dlPFC	R	32, 34, 34	7	6,22	1034	<.001

Note. Clusters listed are significant in a whole-brain analysis ($p<.001$ uncorrected at voxel level and $p < 0.05$ FWE-corrected at cluster level, $k > 20$ voxels). x, y, z refer to MNI coordinates. T refers to the t-score and z the z-score at those coordinates (local maxima). K refers to the number of voxels in each significant cluster. BL refers to bilateral.

Table S7

Whole brain activations for my-turn (MT) during Inclusion > my-turn (MT) during Reinclusion (N=88)

Anatomical Region	Hemisphere	x,y,z	*t*	*z*	*k*	*p*
Putamen	R	20, 10, -8	7,97	6,88	44781	<.001
Lingual gyrus	R	14, -72, -8	7,56	6,61		
Putamen	L	-22, 6, -8	7,27	6,41		
Cerebellum	L	-20, -50, -20	7,19	6,35		
Cuneus	R	10, -82, 32	6,85	6,11		
Superior parietal lobule	R	28, -46, 64	6,84	6,1		
Superior occipital gyrus	L	-14, -90, 32	6,74	6,03		
Cuneus	L	-12, -86, 36	6,74	6,03		
Premotor cortex/Precentral gyrus	R	38, -12, 60	6,67	5,98		
Cerebellum	R	12, -56, -54	6,63	5,95		
Postcentral gyrus	R	60, -18, 24	6,62	5,94		
IFG	L	-54, 14, 8	5,41	5,01	1617	<.001
Temporal pole	L	-56, 8, -10	5,26	4,89		
STG	L	-62, -4, -8	3,92	3,75		
ACC	BL	2, 44, 20	5,16	4,81	417	<.001

Note. Clusters listed are significant in a whole-brain analysis ($p<.001$ uncorrected at voxel level and $p < 0.05$ FWE-corrected at cluster level, $k > 20$ voxels). x, y, z refer to MNI coordinates. T refers to the t-score and z the z-

score at those coordinates (local maxima). K refers to the number of voxels in each significant cluster. BL refers to bilateral.

Table S8

Whole brain activations for Throw during Inclusion > Throw during Reinclusion (N=88)

Anatomical Region	Hemisphere	x,y,z	*t*	*z*	*k*	*p*
dACC	BL	0, 14, 34	6,65	5,96	15476	<.001
SMA	BL	-2, -10, 76	6,11	5,56		
Precentral gyrus	L	-36, -22, 64	6	5,47		
STG	L	-62, -10, 4	5,98	5,45		
IFG	L	-56, 10, 2	5,73	5,27		
Inferior parietal lobule	R	64, -24, 28	5,62	5,18		
Postcentral gyrus	R	48, -26, 56	5,57	5,14		
STG	R	62, -2, -4	5,27	4,9		
PCC	BL	-2, -50, 6	6,31	5,71	5168	<.001
Lingual gyrus	L	-6, -42, 2	5,84	5,35		
Lingual gyrus	R	6, -40, 0	5,53	5,11		
Fusiform gyrus	L	-26, -34, -24	5,18	4,83		
Cerebellum	R	42, -74, -24	4,88	4,58		
Hippocampus	R	38, -8, -20	4,79	4,5		
Amygdala	L	-20, -8, -14	4,71	4,44		
Cerebellum	L	-30, -34, -30	4,65	4,38		
Parahippocampal gyrus	L	-10, -70, -8	4,49	4,25		
Thalamus	R	16, -28, -2	4,45	4,22		
Fusiform gyrus	R	26, -86, -18	4,39	4,16		
Cuneus/ Extrastriate cortex	R	6, -84, 38	6,19	5,62	912	<.001

Cuneus/ Extrastriate cortex	L	-10, -88, 38	5,03	4,7		
Cerebellum	L	-30, -46, -54	5,44	5,03	805	<.001
Cerebellum	R	18, -52, -54	5,3	4,92		

Note. Clusters listed are significant in a whole-brain analysis (p<.001 uncorrected at voxel level and p < 0.05 FWE-corrected at cluster level, k > 20 voxels). x, y, z refer to MNI coordinates. T refers to the t-score and z the z-score at those coordinates (local maxima). K refers to the number of voxels in each significant cluster. BL refers to bilateral.

Table S9

Regions showing significant associations with Parental Involvement in whole brain analysis of the contrast my-turn (MT) during Inclusion versus not-my-turn (NMT) during Exclusion (N=84)

Anatomical Region	Hemisphere	x,y,z	*t*	*z*	*k*	*p*
Cerebellum	R	26, -70, -28	4,91	4,58	1340	<.001
Cerebellum	L	-10, -78, -22	4,59	4,31		
Lingual gyrus	L	-14, -74, 2	4,66	4,38	279	0,038
Lingual gyrus	R	4, -84, -4	3,64	3,50		

Note. Clusters listed are significant in a whole-brain analysis (p<.001 uncorrected at voxel level and p < 0.05 FWE-corrected at cluster level, k > 20 voxels). x, y, z refer to MNI coordinates. T refers to the t-score and z the z-score at those coordinates (local maxima). K refers to the number of voxels in each significant cluster. BL refers to bilateral.

Table S10

Regions showing significant associations with Parental Involvement in whole brain analysis of the contrast my-turn (MT) during Inclusion versus Reinclusion (N=84)

Anatomical Region	Hemisphere	x,y,z	*t*	*z*	*k*	*p*
Cerebellum	L	-44, -54, -26	5,3	4,9	359	0,016
Fusiform gyrus	L	-32, -48, -18	3,53	3,4		
PCC	L	-4, -32, 48	4,64	4,36	301	0,03
PCC	R	4, -34, 46	4,04	3,85		
Cuneus	L	-14, -82, 38	4	3,81	658	0,001
Cuneus	R	2, -84, 32	3,87	3,7		

Note. Clusters listed are significant in a whole-brain analysis (p<.001 uncorrected at voxel level and p < 0.05 FWE-corrected at cluster level, k > 20 voxels). x, y, z refer to MNI coordinates. T refers to the t-score and z the z-score at those coordinates (local maxima). K refers to the number of voxels in each significant cluster. BL refers to bilateral.

Table S11

Regions showing significant associations with Parental Involvement in whole brain analysis of the contrast not-my-turn (NMT) during Inclusion versus Reinclusion (N=84)

Anatomical Region	Hemisphere	x,y,z	*t*	*z*	*k*	*p*
Cuneus	L	-8, -84, 14	4,38	4,14	322	0,025

Note. Clusters listed are significant in a whole-brain analysis (p<.001 uncorrected at voxel level and p < 0.05 FWE-corrected at cluster level, k > 20 voxels). x, y, z refer to MNI coordinates. T refers to the t-score and z the z-score at those coordinates (local maxima). K refers to the number of voxels in each significant cluster. BL refers to bilateral.

Table S12

Regions showing significant associations with Heightened Parenting in whole brain analysis of the contrast my-turn (MT) versus not-my-turn (NMT) during Inclusion for Own Child vs Unrelated Child (N=86)

Anatomical Region	Hemisphere	x,y,z	*t*	*z*	*k*	*p*
Thalamus	L	-22, -20, 8	5,51	5,07	2074	<.001
STG	L	-44, -40, 6	5,22	4,84		
Putamen	L	-24, -4, 0	4,54	4,28		
MTG	L	-56, -44, 8	4,21	4		
Putamen	R	28, -10, -2	5,16	4,79	4295	<.001
dlPFC	R	42, 44, 24	5,04	4,7		
MTG/Frontal eye fields	R	20, 36, 34	4,94	4,62		
Anterior PFC	R	24, 50, -2	4,9	4,58		
Frontal operc. (orbital)	R	30, 6, 30	4,71	4,43		
Insula	R	32, 18, -6	4,69	4,41		
Thalamus	R	18, -10, 2	4,32	4,09		
vmPFC	R	14, 48, 2	4,12	3,92		
IFG (orbital)	R	54, 36, -2	4,1	3,9		
Premotor cortex	R	52, -4, 22	4	3,82		
Anterior PFC	L	-26, 50, 2	4,75	4,45	392	0,011
TPJ/Supramarginal gyrus	R	52, -34, 36	4,66	4,38	634	0,001
Angular gyrus	R	54, -58, 36	3,87	3,7		
MTG	R	38, -64, 10	4,56	4,3	465	0,005

	MTG/Fusiform gyrus	R	44, -68, 2	4,55	4,29		
	STG/Angular gyrus	R	40, -54, 16	3,37	3,26		
SMA		R	12, -20, 66	4,51	4,26	759	<.001
	Precentral gyrus	L	-10, -26, 68	4,18	3,97		
	SMA/Premotor cortex	L	-6, -24, 54	4,1	3,9		

Note. Clusters listed are significant in a whole-brain analysis ($p<.001$ uncorrected at voxel level and $p < 0.05$ FWE-corrected at cluster level, $k > 20$ voxels). x, y, z refer to MNI coordinates. T refers to the t-score and z the z-score at those coordinates (local maxima). K refers to the number of voxels in each significant cluster. BL refers to bilateral.

Table S13

Descriptive statistics for the mean contrasts estimates of mothers (N=40) versus fathers (N=48) for my-turn (MT) versus not-my-turn (NMT) events during Inclusion

Region	Parent Gender	Event type	*M*	*SD*
Putamen	Mothers	My-turn (MT)	1,72	0,17
Cluster (k=304)	Mothers	Not-my-turn (NMT)	0,51	0,12
peak voxel at [-14 14 -4]	Fathers	My-turn (MT)	0,83	0,16
	Fathers	Not-my-turn (NMT)	0,44	0,12
Precuneus	Mothers	My-turn (MT)	1,31	0,11
Cluster (k=491)	Mothers	Not-my-turn (NMT)	0,90	0,10
peak voxel at [14 -58 62]	Fathers	My-turn (MT)	0,78	0,10
	Fathers	Not-my-turn (NMT)	0,80	0,09
dACC	Mothers	My-turn (MT)	0,68	0,12
Sphere 4 mm	Mothers	Not-my-turn (NMT)	0,17	0,09
peak voxel at [10 -12 50]	Fathers	My-turn (MT)	0,37	0,11
	Fathers	Not-my-turn (NMT)	0,38	0,08
MTG/Middle occipital gyrus	Mothers	My-turn (MT)	2,01	0,12
Cluster (k=549)	Mothers	Not-my-turn (NMT)	1,55	0,09
peak voxel at [42 -74 0]	Fathers	My-turn (MT)	1,18	0,11
	Fathers	Not-my-turn (NMT)	1,26	0,11

Table S14

Descriptive statistics for the mean contrasts estimates of mothers (N=40) versus fathers (N=48) for my-turn (MT) during Inclusion versus not-my-turn (NMT) during Exclusion

Region	Parent Gender	Event type	*M*	*SD*
MCC	Mothers	MT Inclusion	1,91	0,17
Cluster (k=362)	Mothers	NMT Exclusion	0,43	0,13
Peak voxel at [0 -14 44]	Fathers	MT Inclusion	1	0,15
	Fathers	NMT Exclusion	0,62	0,11
Dorsal PCC	Mothers	MT Inclusion	0,71	0,18
Sphere 5 mm	Mothers	NMT Exclusion	0,03	0,18
Peak voxel at [0 -34 48]	Fathers	MT Inclusion	0,02	0,17
	Fathers	NMT Exclusion	0,43	0,16
Ventral PCC	Mothers	MT Inclusion	0,13	0,1
Sphere 4 mm	Mothers	NMT Exclusion	-0,06	0,1
Peak voxel at [-10 -46 28]	Fathers	MT Inclusion	-0,13	0,09
	Fathers	NMT Exclusion	0,25	0,1
STG	Mothers	MT Inclusion	4,66	0,28
Cluster (k=278	Mothers	NMT Exclusion	3,89	0,26
Peak voxel at [-60 -26 10]	Fathers	MT Inclusion	3,83	0,25
	Fathers	NMT Exclusion	4,27	0,23
Precuneus	Mothers	MT Inclusion	1,43	0,15
Sphere 5 mm	Mothers	NMT Exclusion	0,59	0,14

Peak voxel at [-6 -44 54]	Fathers	MT Inclusion	0,81	0,14
	Fathers	NMT Exclusion	0,84	0,13
Intraparietal sulcus	Mothers	MT Inclusion	1	0,1
Sphere 5 mm	Mothers	NMT Exclusion	0,18	0,07
Peak voxel at [-30 -40 36]	Fathers	MT Inclusion	0,42	0,09
	Fathers	NMT Exclusion	0,33	0,07
