# Supplementary material for: Caregiver or Playmate? Fathers’ and mothers’ brain responses to ball-play with children
Source: Cogn Affect Behav Neurosci. 2024 Dec 5;25(2):434–53. doi: 10.3758/s13415-024-01237-1 (PMC11906569; doi:10.3758/s13415-024-01237-1)
Supplement: Supplementary file 2 — Supplementary file2 (PDF 112 KB) [file 13415_2024_1237_MOESM2_ESM.pdf]

The neural substrates of social in- and exclusion in parents of pre-school children: a new event-related fMRI study

In this functional magnetic resonance imaging (fMRI) study, we aim at investigating pre-school children's parents' brain activation during a virtual ball ball-toss game ("cyberball"), purportedly played with their own and an unknown child.

Participants

We initially planned to ascertain a total of N=100 (N=50 mothers) participants with complete and good quality data available for the entire project comprising a functional near-infrared spectroscopy (fNIRS) hyperscanning session, two functional tasks and one anatomical (f)MRI scan(s), as well as self-reported and narrative assessment of attachment and caregiving in parents and children. N=91 complete data sets were obtained before COVID-19 related lockdown. As it is unclear whether, and, if yes, when the remaining N=9 data sets can be acquired, data analyses will start with the available N=91 and may be complemented later.

All participants were pre-screened for medical, neurological, and psychological/psychiatric issues, abuse of drugs or alcohol, handedness, biological relatedness of parent and child, reading and writing difficulties, as well as fMRI counter indications. They were furthermore required to be aged 23 to 55. Participants signed a consent form prior to participation and were remunerated. The study was approved by the local ethics committee.

Paradigm & Stimuli

The virtual ball-toss game ("cyberball") programmed using Presentation comprises three main conditions: inclusion, exclusion, and re-inclusion. During inclusion and re-inclusion, all players (i.e. the parent and 2 virtual players parents are led to believe to be their own child and an unknown child) receive the ball equally. During exclusion, the ball is almost exclusively passed between the (computer-generated) children so that parents are excluded from the game.

In comparison to most previous fMRI studies using "cyberball" with a block design averaging all activity across (re-)inclusion and exclusion, our paradigm is tailored to an event-related analysis by imposing static events on a dynamic paradigm (via transient disappearance and reappearance of the ball) that can additionally separate distinct events within (re-)inclusion and exclusion blocks. This means that we can examine brain activity when parents (i) observe children playing with each other ("not-my-turn" events), (ii) catch the ball from children ("my-

turn" events), and (iii) throw the ball to children ("throw" events). Furthermore, we can separate ball-passes between other players ("not-my-turn"), catches ("my-turn"), and throws to the own versus an unknown child. We therefore have three main task conditions (inclusion, exclusion, re-inclusion), three task sub-conditions (not-my-turn, my-turn, throw), and two conditions of familiarity (own child, unknown child).

Pictures of children and parents were taken at the end of the fNIRS session some weeks before the fMRI session, always in front of a light gray background and by wearing a black T-shirt. Parents were asked to pose with a neutral emotional expression. Children were filmed for 2-5 minutes while posing with a neutral, happy, and angry facial expression. From child film clips, three displays per emotion were selected and rated by three independent raters upon which one display per emotion was selected. After the fMRI session, every parent furthermore rated their own and the unknown child's emotional displays on several criteria. Unknown children were randomly paired with own children in a gender-matched fashion.

fMRI Data Analysis

fMRI data is being pre-processed using a standard SPM pipeline. We are deriving individual models on the 1st level by creating regressors for "not-my-turn", "my-turn" and "throw" events involving the own versus an unknown child for each of the three main conditions. Because during exclusion, "my-turn" and "throw" events occur very rarely, these cannot be reliably modelled as individual regressors/conditions. However, these trials are nonetheless included as an additional regressor. Furthermore, any missed or wrong trials are also added to this additional regressor so that every single trial is included in the model.

Main Effects

In a first step, whole-brain event-related group analyses will focus on the following main contrasts:

- Comparison of "not-my-turn" events for inclusion versus exclusion conditions. Subsequent analysis for possible effect of familiarity (own versus unknown child).
- Comparison of "not-my-turn" events for inclusion versus re-inclusion conditions. Subsequent analysis for possible effect of familiarity (own versus unknown child).
- Comparison of "my-turn" events for inclusion versus re-inclusion conditions. Subsequent analysis for possible effect of familiarity (own versus unknown child).
- Comparison of "throw" events for inclusion versus re-inclusion conditions. Subsequent analysis for possible effect of familiarity (own versus unknown child).

In a second step, whole-brain event-related group analyses will focus on the following contrasts:

- Comparison of “my-turn” versus “not-my-turn" events within the inclusion condition. Subsequent analysis for possible effect of familiarity (own versus unknown child).
- Comparison of “my-turn” versus “not-my-turn" events within the re-inclusion condition. Subsequent analysis for possible effect of familiarity (own versus unknown child).
- Comparison of “my-turn” versus “not-my-turn" events for inclusion versus re-inclusion conditions. Subsequent analysis for possible effect of familiarity (own versus unknown child).

In subsequent exploratory analyses, we will assess the potential influence of parent and child biological sex on above contrasts. We may also look at possible associations between brain activity and other demographic variables such as parent age, education, and family income, as well as individual image ratings provided by parents after the fMRI session. All participants furthermore filled in a dedicated “cyberball” / social exclusion questionnaire after the fMRI session. This measure (particularly the question of how real the parents thought the “cyberball” was) may also be analyzed and potentially included as a control variable in whole-brain contrasts.

Finally, we will compute additional exploratory contrasts for purposes of comparison with previous work.

All whole-brain analyses will use a combined statistical threshold of $p < .001$ uncorrected at the voxel level with $k < 20$ and $p < .05$ FWE-corrected at the cluster level.

Outliers and Exclusions

Outliers in demographic and rating values will be defined as values above or below 3 standard deviations from the mean. These values will be winsorized to the respective upper or lower boundaries (if not clearly representing measurement errors, which will be removed).

fMRI data will be excluded from analysis if showing too many/strong motion artefacts during pre-processing (especially continuous motion exceeding 2.5 mm in x, y, and/or z translation – voxel size is 2.5 mm^3), prominent signal loss after visual inspection, and/or too many erroneous trials (missed responses).
